# Multimodal Integration of M/EEG and f/MRI Data in SPM12

**DOI:** 10.3389/fnins.2019.00300

**Published:** 2019-04-24

**Authors:** Richard N. Henson, Hunar Abdulrahman, Guillaume Flandin, Vladimir Litvak

**Affiliations:** ^1^MRC Cognition and Brain Sciences Unit, University of Cambridge, Cambridge, United Kingdom; ^2^Wellcome Centre for Human Neuroimaging, University College London, London, United Kingdom

**Keywords:** MEG, EEG, fMRI, multimodal, fusion, SPM, inversion, faces

## Abstract

We describe the steps involved in analysis of multi-modal, multi-subject human neuroimaging data using the SPM12 free and open source software (https://www.fil.ion.ucl.ac.uk/spm/) and a publically-available dataset organized according to the Brain Imaging Data Structure (BIDS) format (https://openneuro.org/datasets/ds000117/). The dataset contains electroencephalographic (EEG), magnetoencephalographic (MEG), and functional and structural magnetic resonance imaging (MRI) data from 16 subjects who undertook multiple runs of a simple task performed on a large number of famous, unfamiliar and scrambled faces. We demonstrate: (1) batching and scripting of preprocessing of multiple runs/subjects of combined MEG and EEG data, (2) creation of trial-averaged evoked responses, (3) source-reconstruction of the power (induced and evoked) across trials within a time-frequency window around the “N/M170” evoked component, using structural MRI for forward modeling and simultaneous inversion (fusion) of MEG and EEG data, (4) group-based optimisation of spatial priors during M/EEG source reconstruction using fMRI data on the same paradigm, and (5) statistical mapping across subjects of cortical source power increases for faces vs. scrambled faces.

## Introduction

As part of this Special Research Topic on how to perform MEG/EEG group analysis with free academic software, we describe practical steps using the SPM12 software package (https://www.fil.ion.ucl.ac.uk/spm/) and a publically-available multimodal dataset. We describe SPM's graphical user interface (GUI), its “batch” interface for linear pipeline creation and finally “scripting” in MATLAB for (parallelised) loops across subjects.

The paper is organized into sections with a brief theoretical background followed by a detailed step-by-step walkthrough. The background is only brief because we refer to previous published papers, many of which are available from the SPM website: https://www.fil.ion.ucl.ac.uk/spm/doc/biblio/. We do not provide a full tour of all the available options in SPM for M/EEG, which is already present in Litvak et al. (2011). Rather, we focus on a single, typical pipeline for creating event-related responses, localizing those responses in the brain and performing statistics on the results. Our experience with teaching SPM is that students appreciate having a concrete example, which they can then adjust to their own needs^1^.

For an overview of the dataset see, Wakeman and Henson (2015). The data are in BIDS format, both MRI (Gorgolewski et al., 2016) and MEG (Niso et al., 2017), on the OpenNeuro platform: https://openneuro.org/datasets/ds000117/versions/ 1.0.2^2^.

The MEG data consist of 102 magnetometers and 204 planar gradiometers from an Elekta VectorView system. The same system was used to simultaneously record EEG data from 70 electrodes (using a nose reference), which are stored in the same “FIF” format file (as well as bipolar horizontal and vertical electro-oculograms, HEOG/VEOG, and bipolar electro-cardiogram, ECG). The data include a raw FIF file for each run/subject, but also a second FIF file (see below) in which the MEG data have been cleaned using Signal-Space Separation (Taulu et al., 2004) as implemented in MaxFilter 2.1 (Elekta Neuromag; https://accessgudid.nlm.nih.gov/devices/06430056480046). We use the latter here. A Polhemus digitizer was used to digitize three fiducial points and a large number of other points across the scalp, which can be used to coregister the M/EEG data with the structural MRI image. Six runs of ~10 min were acquired for each subject, while they judged the left-right symmetry of each stimulus, leading to nearly 300 trials in total for each of the 3 conditions (famous face, unfamiliar face, scrambled face).

The MRI data were acquired on a 3T Siemens TIM Trio, and include a 1 × 1 × 1 mm T1-weighted structural MRI (sMRI) as well as a large number of 3 × 3 × ~4 mm T2^*^-weighted functional MRI (fMRI) EPI volumes acquired during 9 runs of the same task (performed by same subjects with different set of stimuli on a separate visit). Note that the T1 images have had the face removed to protect the identity of the subjects (non-de-faced images, e.g., for more accurate head-modeling, are available from a subset of subjects on request to rik.henson@mrc-cbu.cam.ac.uk). Other data on the same subjects, such as ME-FLASH and Diffusion-Weighted images, plus empty-room MEG data, are available on the OpenNeuro site, which could be used for improved head modeling and source localization, but are not used here.

Each analysis step is a separate SPM batch module. The batch interface is a generic GUI in SPM that allows configuring and running complex analyses without programming. This interface can be used to thread together multiple modules to create a linear pipeline. When we want to repeat that pipeline across multiple runs or multiple subjects, we can save it as a batch script, and use some simple MATLAB commands to loop over runs/subjects, just by changing the input files to the pipeline. Finally, for the more advanced user (familiar with the MATLAB syntax), we also provide a script (see below) that runs the full analysis from start to finish by direct calls to SPM12 MATLAB functions (without necessarily using the batch interface).

It should be noted that the pipeline described below is just one possible sequence of processing steps, designed to illustrate many of the options available in SPM12. It is not necessarily the optimal preprocessing sequence, which really depends on the question being asked of the data.

## Getting Started

Download the data in BIDS format from OpenNeuro, e.g., to **/yourpath** (we will call this the “rawpth”)^3^. The entire dataset is around 170GB so we suggest to have at least 300GB available for the entire analysis pipeline. If you need to save space, you can delete all the sub-^*^/ses-mri/anat/^*^FLASH.nii.gz and sub-^*^/ses-mri/dwi/^*^_dwi.nii.gz files, as we do not use them here.

The analyses described below require SPM12, a free and open source software developed at the Wellcome Center for Human Neuroimaging and available for download at:

https://www.fil.ion.ucl.ac.uk/spm/software/download/

It runs under MATLAB and is compatible with all versions between R2007a and R2019a, on Linux, Windows and macOS. Installing SPM12 only requires unzipping the archive and adding the main directory to the MATLAB path^4^. For the analyses here, we use SPM12 r7487 released in November 2018.

Next, you should create a sub-directory called “**code**” within **/yourpath**, into which you should unzip all the scripts and batch files downloadable from Figshare:

https://figshare.com/collections/Multimodal_integration_of_M_EEG_and_f_MRI_data_in_SPM12/4367120.

This **code** directory includes two sub-directories: (1) one called “**manual**”, which contains copies of all the SPM batch job files that will be created below (as well as a master script to link them together called “**batch_master_script.m**”), and (2) another called “**scripted**”, which contains a “**master_script.m**” that illustrates instead direct calls to spm^*^.m functions (bypassing the batch system, except for a few exceptions), which can be run to reproduce all the results in this paper, including the figures, which can be reproduced by the additional script “**create_figures.m**”.

First, you also need to create a directory for SPM's output, which we will call “**outpth**”, e.g.:

**/yourpath/derivatives/SPM12**

You can then create sub-directories for all subjects using some SPM/MATLAB code like: 
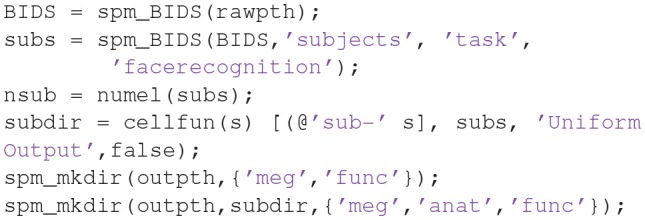


Finally, you will also need to copy and unzip all the raw MEG and sMRI files to **outpth** (since this is where SPM will write all files derived from them), which you can do with the following code (this code is also present at the start of the **master_script.m**): 
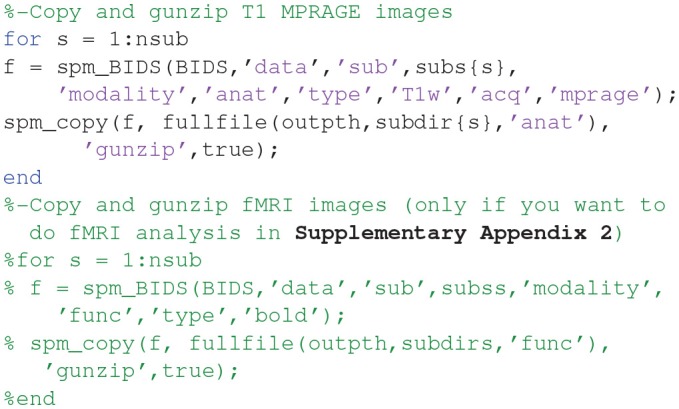


Note that if you want to run the fMRI analysis (e.g., following Supplementary Appendix 2, or using the **master_script.m**), you will need to uncomment the last section of code above. However, in case you want to save time and disk space, we also provide the results of that fMRI analysis (which is needed for the fMRI-informed source-localization of M/EEG data described in section Group and fMRI Optimized Source Reconstruction) on the above Figshare link.

**Figure 1:**
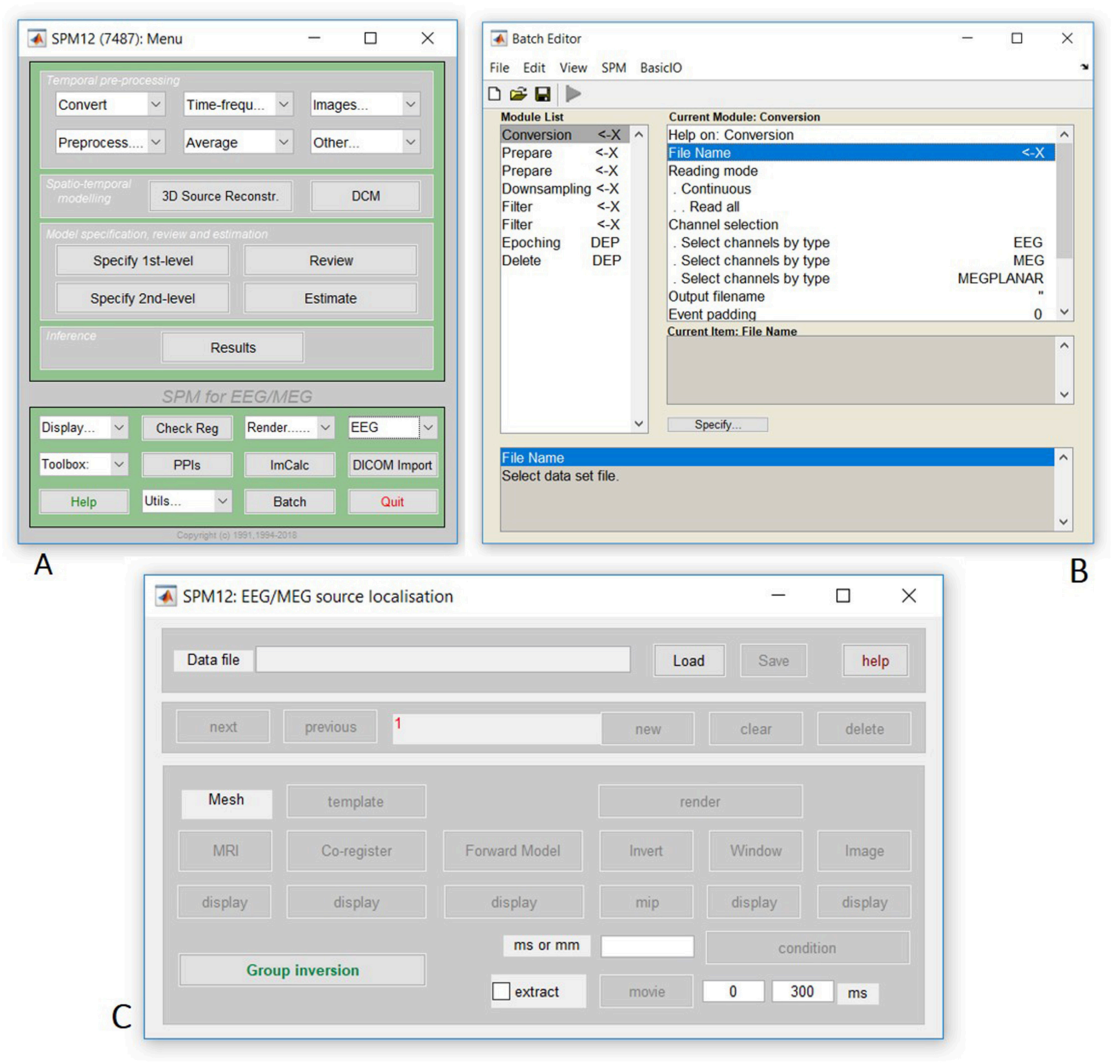
Screenshot of SPM figures **(A)** SPM main menu, **(B)** SPM batch interface, **(C)** Source localization (reconstruction) interface.

Finally, open the SPM12 graphical interface by typing “**spm eeg**” at the MATLAB prompt, which should open three windows (including that in Figure 1A). Then open the batch editor window by pressing “**Batch**” from the SPM: Menu window, which should open the window in Figure 1B. (Later we will press the “**3D Source Reconstruction**” button to get the window in Figure 1C).

## Preprocessing M/EEG Data

### Motivation and Background

The first aim of pre-processing is to transform the data from the format originally recorded in the scanner (which varies across scanner types) to a common format used by SPM (and closely related to that used by FieldTrip). A second aim is to perform some basic operations on the data like filtering, epoching and removal of non-interesting artifacts. The resulting “cleaned” data can then form the input to advanced analyses in SPM, such as statistical parametric mapping, source reconstruction and Dynamic Causal Modeling (though the latter is not discussed in the present paper). In principle, pre-processing in SPM is not different from that in other academic and commercial M/EEG analysis software packages. Therefore, data could also be fully or partially pre-processed outside of SPM as long as the results are converted to SPM format. Here we show full pre-processing in SPM with the exception of Signal Space Separation, which is done in manufacturer's software as previously mentioned.

The order of steps shown here is just one of many possibilities. Depending on the specifics of your own data, you might choose to arrange the steps differently. However, the following are some points to remember when designing a preprocessing pipeline in SPM:

- In the present example, we will convert the data as a continuous timeseries, though it is possible to “cut out” time windows (epochs) around the trial onsets during the conversion step, e.g., if you wanted to save disk space and processing time.
- Digital filtering might create artifacts (ringing) where there are discontinuities in the data, particularly at the edges. It is, therefore, better to filter continuous data prior to epoching to avoid filter ringing artifacts in every trial. Alternatively the epochs of interest can be padded with more data and then cropped after filtering.
- Since the ringing depends on the amplitude of the discontinuity, it is better to do high-pass filtering or baseline correction before other filtering steps.
- It is convenient to put downsampling early in the pipeline to make the subsequent steps faster.
- SPM only filters channels with physiological data. So the channel types should be set correctly before filtering.
- Some artifacts (e.g., discontinuous jumps or saturations) are more difficult to detect after filtering. In SPM, there is an option to mark artifacts in continuous data and use this information later in the pipeline e.g., for trial rejection, but we do not consider that here.
- One common distinction is whether analyses are performed over time (e.g., evoked response amplitudes), over frequency (e.g., power and/or phase after Fourier transform), or time-and-frequency (e.g., using wavelets). Below we illustrate a typical time-based analysis of evoked responses, i.e., event-related potentials (ERP) from EEG and event-related fields (ERF) from MEG. In Supplementary Appendix 1, we also illustrate an alternative analysis using wavelets to capture both evoked and induced power.

### Tutorial Walkthrough

**Figure 2:**
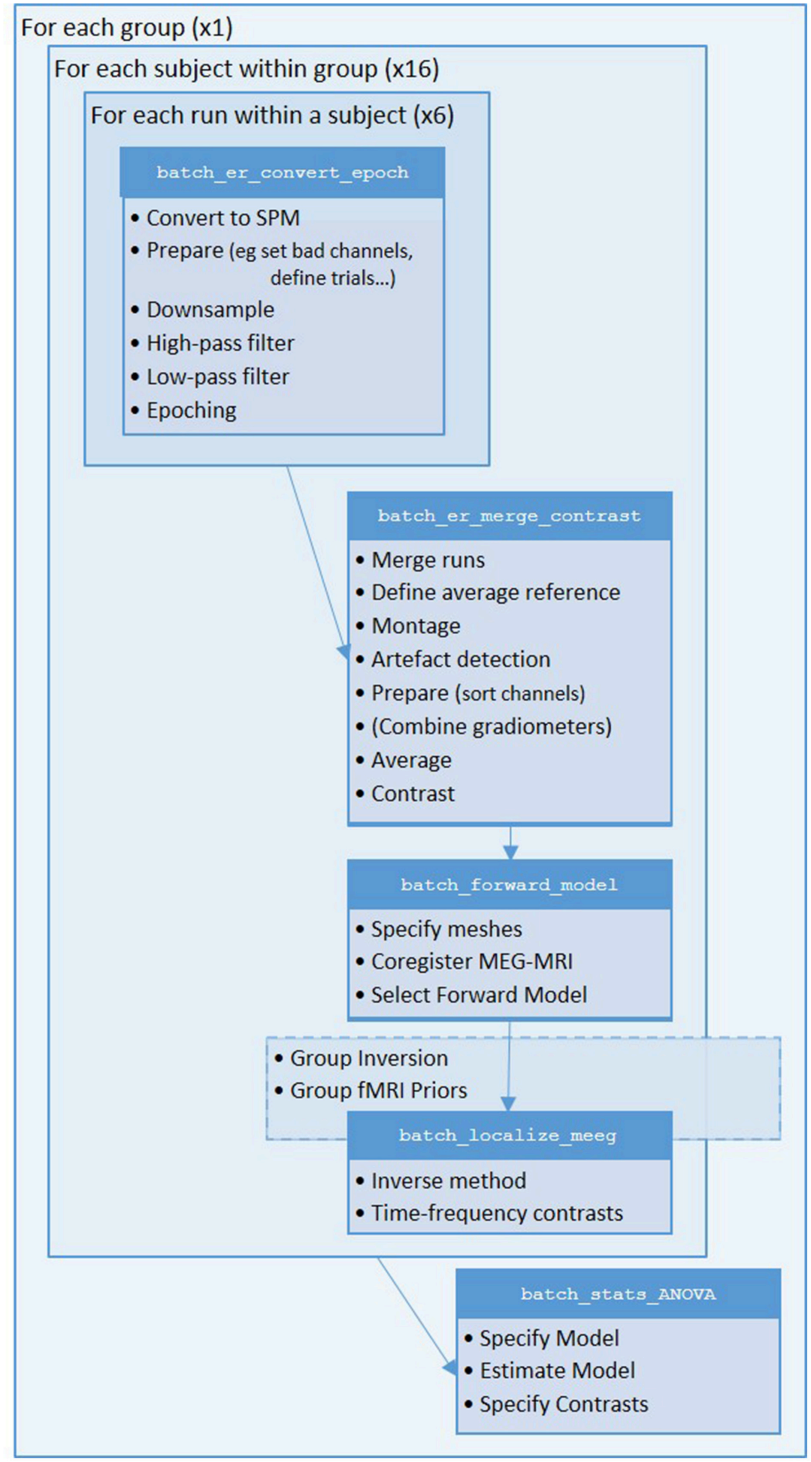
Full pipeline. The MATLAB filenames at the top of each box refer to the batch files (in the “manual” directory) used for each step.

We will start by creating pipelines (using SPM's batch interface) for preprocessing the M/EEG data for a single subject, and then scripting these pipelines to repeat over multiple subjects. We will start with Subject 15, in whom the data are particularly clean. The full preprocessing pipeline is shown in Figure 2.

#### Convert

The first step is to convert raw M/EEG data from its native format (which depends on the acquisition system) to the MATLAB format used by SPM.

In the batch editor, select SPM on the top toolbar, and from the dropdown menu select **M/EEG**. At the top of the new dropdown menu, select “**Conversion**”. Once selected, the Module List on the left of the batch editor window will now list Conversion as the first (and only) step. Within the main, Current Module window will list several variables. The first variable listed is File Name. On the right hand side of this pane, you will see “**<-X**”; this indicates that you need to update this field. To do so, click on **File Name**, which will then open up your current working directory. Select the file named “**sub-15_ses-meg_task-facerecognition_run-01 _proc-sss_meg.fif**” in the “**outpth/sub-15/meg**” directory and press “**done**”.

Many variables in the Current Module window have default values, but we need to change some of them. For example, we do not want to epoch during conversion, leave the default “**continuous**” option; we can epoch the data later using another SPM module.

Another change to the defaults is that we do not want to convert all channels in the original file (since many are extraneous), so will select a subset by their type. We first need to delete the default option to convert all channels. To do this, click “**channel selection**”, and scroll down until you can select the “**Delete All(1)**” option. Then click the “**channel selection**” again, but this time choose the option “**New: Select channels by type**”. This will add “**Select channels by type**” to the Current Module, and you will see “**<-X**” on the right hand side of this, indicating the need for user input. Click the “**<-X**” and then select “**EEG**” from the “**Current Item**” section. Repeat this process to additionally include the “**MEG**” and “**MEGPLANAR**” channels.

Finally, we want to read in the stimulus trigger channel, which for this dataset is called “**STI101**”. Note that you do not need to read in this channel when you use the event definitions provided by BIDS (we will use these BIDS definitions later). But to illustrate first how you could define those events yourself based on a trigger channel, we will include this channel. Click the “**channel selection**” again, but this time choose the option “**New: Custom channel**”. Select the new “**<-X**” that appears and specify “**STI101**” as the value.

The remaining options for conversion can be left with their default values (which includes the output filename, which defaults to the input filename, prepended with “**spmeeg_**”). Once all variables are specified, the play button on the top toolbar will turn green and the batch could be run. However, for this example, we will continue to use the current batch editor window, so do not press the play button yet.

#### Prepare (Define Channels)

The next step in the current pipeline is to update some other properties of the data using the “**Prepare**” module. This is a general-purpose “housekeeping” module that includes options like re-defining channel names, types, locations, etc. as specific to the particular laboratory set-up. In our case, some of the channels currently labeled EEG were in fact used to record EOG.

Select “**Prepare**”, from the preprocessing menu. Highlight “**Prepare**” in the Module list; this will open up the variables in the current module window. Again we need to complete those variables indicated by “**<-X**”. If we had already run the previous conversion stage, we could select the new “**spmeeg_sub-15_ses-meg_task-facerecognition _run-01_proc-sss_meg.mat**” file produced by that stage as the input for this stage. Here however, we will create a pipeline in which all stages are run in one go, in which case we need to tell SPM that the output of the conversion step, even though not yet created, will be the input of this preparation step. You can do this by selecting the “**Dependency**” button located further down the window. This will open up a new window, listing all the processing steps up to this point. So far this is just one: the conversion step. Highlight this step and select “**OK**”.

The next variable to define is the “**Select task(s)**”. Clicking this variable will display a variety of options in the “current item” box. Within this, select “**New: Set channel types from BIDS**”, and then select the file “**task-facerecognition_channels.tsv**” in the main BIDS directory. This file contains meta-information about channels, including the fact that, for the specific MEG laboratory from which these data were acquired, channel EEG061 was actually HEOG, channel EEG062 was VEOG, channel EEG063 was ECG and channel EEG064 was unused (free-floating, so should be ignored).

Now create a second “**Prepare**” module, but this time select the “**New: Set bad channels from BIDS**” task, and again select the channel file “**task-facerecognition_channels.tsv**”. Then select the previous “**Prepare**” module as the dependency for input. This will update the data with those channels that were marked as “bad.” Actually, there were no bad channels marked for this dataset, but we include this step for a more generic pipeline, e.g., you could create a separate “^*^channels.tsv” file for each subject and mark channels that you think are bad. Note also that there are many other ways that bad channels can be defined automatically by SPM (or other MATLAB toolboxes such as those from FieldTrip), but these options are not explored here.

#### Define Trials

The onset of trials (events) are normally defined by codes sent from a stimulus machine to the MEG device, which are recorded in a trigger channel (which is channel STI101 in the present data). SPM has some ability to define trials from that channel, which we will illustrate in a brief digression. But for the main pipeline, we will read the trial definitions from a BIDS file instead, because in some subjects and runs, the trigger channel had a complex mixture of stimulus and key codes, and more generally, a trial-type may be defined by complex rules involving a combination of multiple triggers (e.g., when “correct trials” are defined as a specific stimulus code followed by a specific key code). Such complex and bespoke rules are beyond SPM's capabilities, so require the experimenter to define the trial onsets themselves.

##### Defining trials from trigger channel

If you want to try defining trials from the trigger channel, then you would add a new “**Epoching**” module. Select the output from the last “**Prepare**” module as the input dependency, and specify “**Define trial**” under the next “**How to define trials**” option. For “**Time window**”, enter [−100 500], for the start of prestimulus period and end of epoch (in ms). Then under “**Trial definitions**”, select “**New: Trial**” and enter “Famous” as the “**Condition label**”, “STI101_up” as the “**Event type**”, [5 6 7] as the “**Event value**” and “34” as the “**Shift**”. These choices tell SPM that the onset of Famous trials start when the trigger channel first reaches a value of 5, 6, or 7 (usually from a baseline value of 0)—since the trigger is often a top-hat pulse that lasts several samples. The trigger values are arbitrary, and defined by the experimenter. The 34 ms shift is because there is a delay of 2 screen refreshes at 60 Hz between the trigger pulse from the stimulus machine and when the visual projector actually presented the stimulus to the subject (this will depend on the MEG lab, and can be calibrated with a light diode).

Then select the “**Replicate: Trial(1)**” twice, and for the second “**Trial**”, change the “**Condition label**” to “Unfamiliar” and “**Event**” values to [13 14 15], and for the third “**Trial**”, change the “**Condition label**” to “Scrambled” and the “**Event**” values to [17 18 19].

##### Defining trials from BIDS file

For the main batch below, we will read the trials from a BIDS file, rather than the trigger channel, which we can do via yet another “**Prepare**” module. This will update the trial information within SPM's data structure, which will be used when the “**Epoching**” module is called later (in section Epoch, after downsampling and filtering, which are operations best done on continuous rather than epoched data).

In the new “**Prepare**” module, select the output from the last “**Prepare**” module as the input dependency, select the task “**Load events from BIDS tsv file**”, and then select the file “**sub-15_ses-meg_task-facerecognition_run-01 _events.tsv**” (keep the default option of replacing previous trial definitions in file with these new BIDS ones). Now we can proceed to downsampling the continuous data.

#### Downsample

The data were sampled at 1,100 Hz, but for the analyses below, we rarely care about frequencies above 100 Hz. So to save processing time and disk space, we can downsample the data to 200 Hz (which includes a lowpass filtering to avoid aliasing). Select “**Downsample**” from the module list, click on “**File Name**”, select “**Dependency**” and in the pop-up window, select the prepared datafile at the bottom of the list. Next, set the downsampling rate by clicking the “**New sampling rate**” variable within the current module box. Type “200” into the pop-up window that appears and use “**OK**” to set this value. The output file of this stage will be prepended with a “**d**”.

**Figure 3:**
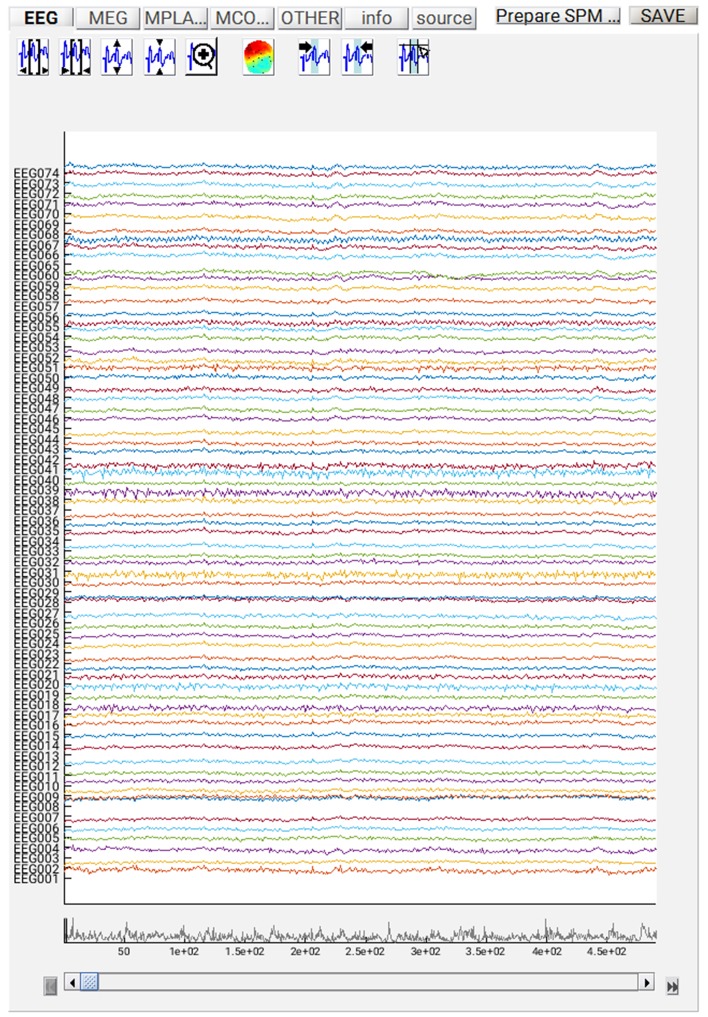
Continuous EEG recordings in SPM graphics window.

If you want to review the continuous data in SPM, you can execute the steps so far by pressing the play button, and when it has finished, press “**Display**” on the main SPM menu, select “**M/EEG**”, then select the file “**dspmeeg_sub-15_ses-meg_task-facerecognition _run-01_proc-sss_meg.mat**”. SPM's Graphics window should show the “**Info**” tab describing the steps done so far, the channels etc. If you select the “**EEG**” tab on the left, then you should see the EEG channels as in Figure 3 (which you can scroll through using slider at the bottom and change the scale etc. using icons at the top, and you might need to change the data scaling, e.g. press the fourth “downscale” button on top right of window, to exactly match Figure 3 below). Once you have finished reviewing the data, go back to the batch window so we can add some further processing steps (modules) that need to be performed on each run, before we explain how to script a loop over runs.

#### Filter

Next, we want to remove low-frequency noise in the data by using a high-pass filter. Go to **SPM->M/EEG->Preprocessing** and add the “**Filter**” Module. Then from the current Module window, use “**Dependency**” to add the output of the previous, downsample module, leave “**Type**” as the default “Butterworth,” and change the “**Band**” to “Highpass”. For the “**Cutoff(s)**”, enter 1 (Hz) and leave the “**Direction**” variable as “Zero phase” and the “**Order**” as 5. Note that “Zero phase” in this case means that the 5th order filter is applied in a two-pass manner, resulting in an attenuation that corresponds to what would have been achieved using a one-pass filter with double the order (i.e., 10). The output file of this stage will be prepended with an “**f**”. We will additionally low-pass filter the data, to remove high-frequency noise (for event-related analyses)^5^. To do this, right click on the “**Filter**” in the Module List and select “**replicate**”. Change “**Band**” to “Lowpass” and enter 40 (Hz) for the “**Cutoff(s)**”, leave everything else as the default, except to update the input dependency to now be the output of the previous filter module above. This will prepend a second “**f**”. The filter type and order used here are the defaults in SPM. They normally work well for cases when there is no special concern about preserving the shape or latency of response peaks. The main advantage of Butterworth filters is that they have relatively little passband and stopband ripple. So noise close to the cut-off frequency cannot inadvertently be amplified by the filter. We refer the interested reader to Widmann et al. (2015) for a detailed discussion of filter design for electrophysiological data.

#### Epoch

In section Define Trials, we inserted the trial onsets and types from the BIDS events file into the SPM object. We now need to use these definitions to cut the continuous data into a number of epochs, one per trial. Select the filtered file from the previous step as the dependency for the input, and then press “**Define Trial**” for “**How to define trials**”. For the “**Time window**”, enter [−100 500], which corresponds to an epoch that starts 100 ms before the stimulus onset and stops 500 ms after. Then on “**Trial definitions**”, select “**New:Trial**” and enter “Famous” as the “**Condition label**”, “BIDS” as the “**Event type**” and “‘Famous”' as the “**Event value**”. Note the single quotes around the event value (to match the value given by the BIDS events file). Leave the “**Shift**” as 0 (because the times in the BIDS file have already been corrected for the 34 ms delay between trigger and stimulus appearing on screen described in section Define Trials).

Then select the “**Replicate: Trial(1)**” twice, and for the second trial, change the “**Condition label”** to “Unfamiliar” and “**Event value**” to “‘Unfamiliar',” and for the third trial, change the “**Condition label**” to “Scrambled” and the “**Event value**” to “‘Scrambled”' (not forgetting the single quotes). For the “**Baseline correction**” option, select “No.” This is because the high-pass filtering above will remove most of the signal drifts that baseline correction is normally used for. Note, however, that there is still a lively debate about whether baseline correction or high-pass filtering is a better method. The output from this step will be prepended with “**e**”.

#### Delete Intermediate Steps (Optional)

The four steps (modules) described above create a preprocessing pipeline for the data. If this pipeline is run straight away, there will be four new files output. If you are short of disk space, you might want to delete some of the intermediate files. To do this, select “**SPM**” from the top toolbar of the batch editor window and choose “**M/EEG –> Other –> Delete**” several times. Then you will need to specify the **File Names** to delete. Highlight each “**Delete**” module and set the **File Name** as the output of the “**Prepare**” step using the “**Dependency**” button to delete any output from the conversion/prepare step onward. However, do not delete the most recent step (epoching), which we need below, nor should you delete the downsampled file, because that will be the starting point for the alternative time-frequency analysis in Supplementary Appendix 1.

##### Create a script for combining pipelines within a subject

Once you have created a linear pipeline, you might want to repeat it on multiple runs (sessions) within a subject, or even across multiple subjects. In the present case, there were 6 independent MEG runs (separated only by a short period to give the subjects a rest), which can all be processed identically. One option would be to save the batch file, manually alter the “**File Name**” that is initially loaded into the batch editor, run it, and repeat this process separately for each run. A more powerful approach is to create a script.

To do this, we first need to remove the files specific to Run 01 above. In the batch window, select the “**Conversion**” task, then right-mouse on “**File Name**” and choose “**Clear Value**” (and the “**<-X**” should return). Repeat this right-mouse clearing of values on three remaining inputs: the “**Set channel types from BIDS tsv**” in the second and third “**Prepare**” modules, and the “**Load events from BIDS tsv file**” input in the fourth module^6^.

Now select **File** from the Batch Editor window, and select “**Save Batch and Script**”. This will produce two files: a batch file (same as that created when you save a batch) and also a MATLAB script that calls that batch file. So if you call the batch file “**batch_er_convert_epoch**”, you will get a batch file called “**batch_er_convert_epoch_job.m**” and a script file called “**batch_er_convert_epoch.m**” (see prepared examples in “**manual**” sub-directory that you downloaded into the “**code**” directory earlier).

The script file “**batch_er_convert_epoch.m**” will automatically be loaded into the MATLAB editor window, and should appear something like this: 
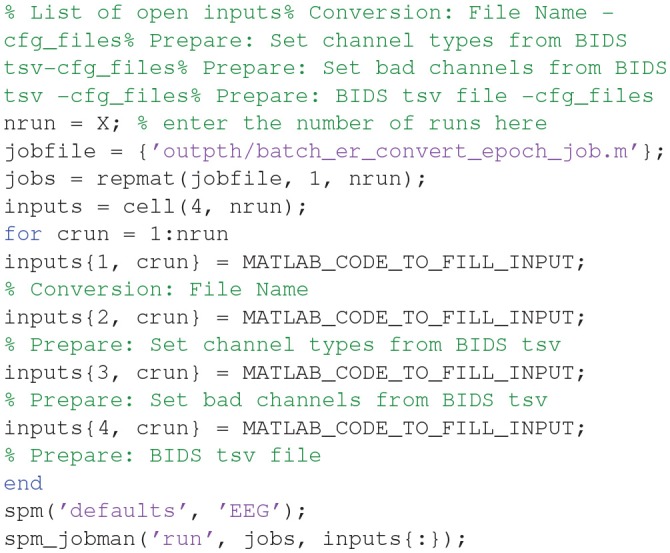


At the top of this script is listed the variable “nrun = X;”. Replace X with 6 for the six runs you wish to convert. You also need to complete the missing MATLAB code needed for each run: (1) the raw input ^*^.fif file to convert for that run, (2) the BIDS ^*^.tsv channel file (for the channel types), (3) the BIDS ^*^.tsv channel file (for the bad channels), and (4) the BIDS ^*^.tsv events file for that run. In order to automate selection of these files, you need to know some basic MATLAB. For example, because the BIDS files are named systematically, we can complete the relevant lines of the above script with: 
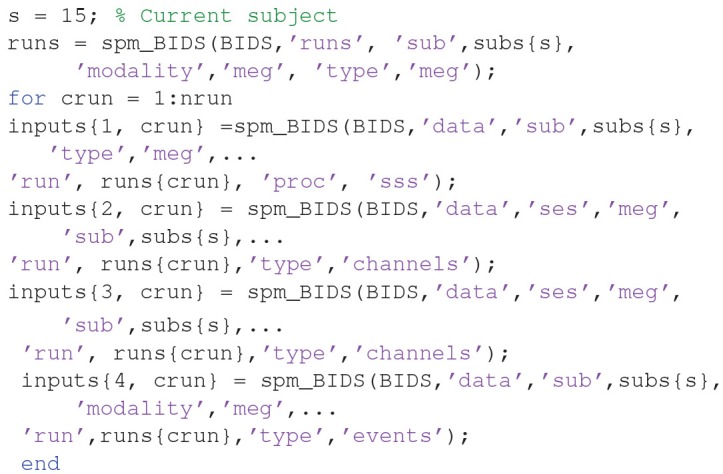


This completes the first part of the preprocessing pipeline. You can then run this script by selecting the green play button on the upper toolbar of the script MATLAB Editor window. The results will be 6 files labeled “**effdspmeeg_sub-15_ses-meg_task-facere cognition_run-%02d_proc-sss_meg.mat**”, where %02d refers to the run number 1–6 (with 0 in front). If you want to view any of these output files, press Display on the main SPM menu pane, select “**M/EEG**”, then select one of these files. You will be able to review the preprocessing steps as a pipeline from the “**History**” section of the “**Info**” tab, and can view single trials by selecting one of the EEG, MEG (magnetometer) or MPLANAR (gradiometer) tabs.

#### Merge (Concatenate Runs)

To analyse the data as one file, the six runs need to be merged. To do this, select “**Merging**” from “**SPM –> M/EEG –> Preprocessing –> Merging**”, select “**File Names**”, “**specify**”, and select the 6 file names “**effdspmeeg_sub-15_ses-meg_task-facerecogn ition_run-%02d_proc-sss_meg.mat.**” If you were to run this stage now, the output file would match the first input file, but be pre-pended with a “**c**”, i.e., “**ceffdspmeeg_sub-15_ses-meg_task-facerecogn ition_run-01_proc-sss_meg.mat**”. However, we will wait to add some more modules before running, as below. At this stage, you could also add “**Delete**” modules to delete all the previous individual run files (since the concatenated file will contain all trials from all runs, i.e., contain the same data).

#### Prepare (Montage for EEG Re-referencing)

First, we want to re-reference the EEG data to the average across channels (as is sometimes conventional for ERP analyses; note the MEG data have no reference). We can do this with the “**Montage**” module below, which is a general purpose module for creating new channel data from linear combinations of existing channel data. However, we first need to create a montage file, which includes a matrix that, when multiplied by the existing data, creates the new channel data. There is another sub-function (task) of the Prepare module that does this, so add another “**Prepare**” module, select the dependency on the previous merged file as the **FileName**, but for the “**task**”, select “**Create average reference montage**” and enter “**avref_montage.mat**” as the output filename. (If you want to look at this montage, you can run this module, load “**avref_montage.mat**” into MATLAB and look at the “**montage.tra**” matrix, where you can see that each new EEG channel is equal to the old EEG channel minus the average of all other EEG channels).

#### Montage

Now we have the montage file, we can apply it, in order to re-reference the EEG data to the average. Select “**Montage**” from the Preprocessing menu, and specify the “**File Name**” as being dependent on the output of the “**Merge**” module above. For the “**Montage file name**”, choose a different dependency, namely the output of the “**Prepare**” module above. Next, highlight “**keep other channels**” and select “yes” in the “**Current Item**” box, in order to keep all the MEG channels (which are unchanged). All other default values can remain the same. The output file will be prepended with “**M**”.

#### Artifact Detection

There are many ways to define artifacts (including special toolboxes; see other SPM manual chapters). Here we focus on just one simple means of detecting blinks by thresholding the EOG channels. Select “**Artifact detection**” from the “**SPM –> M/EEG –> Preprocessing**” menu. For the input file, select a dependency on the output of the previous step. Next, select “**New: Method**” from the box titled “**Current Item: How to look for artifacts**”. Back in the “**Current Module**” window, highlight “**Channel selection**” to list more options, choose “**Select channels by type**” and select **EOG**. Then do not forget to also delete the default “**All**” option! Then press the **<-X** to select “**threshold channels**”, click the “**Specify**” button and set this to 200 (in units of microvolts). The result of this thresholding will be to mark a number of trials as “**bad**” (these can be reviewed after the pipeline is run if you like). Bad trials are not deleted from the data, but marked so they will be excluded from averaging below. The output file will be prepended with the letter “**a**”.

#### Sort Conditions

At this point, we can also do one more bit of house-keeping within the same “**Prepare**” module, which is simply to re-order the condition labels. This only matters for the final stage of “**Contrasting conditions**” below, where the contrast weights assume a certain order of the conditions. The current order of conditions is based purely on the order they appear in the raw data (e.g., if the first few trials of the first run were: “Scrambled, Unfamiliar, Unfamiliar, Scrambled, Familiar…,” then the condition labels will be ordered “Scrambled-Unfamiliar-Familiar”), and this may vary across subjects. To set the condition order to be invariant across subjects, add a new task by selecting the “**Sort conditions**” task, then “**Specify conditions lists**” add three “**New: Condition labels**”, and name them “Famous,” “Unfamiliar” and “Scrambled” (in that order). Note that this operation does not physically reorder the trials at this stage, but just defines the order that will be used where required at later steps.

#### Combine Planar Gradiometers

The next step is only necessary for scalp-level analyses on planar gradiometers, but we include for completeness (see https://www.fil.ion.ucl.ac.uk/spm/doc/manual.pdf#Chap:data:multi for example of scalp-time statistics). Neuromag's planar gradiometers measure two orthogonal directions of the magnetic gradient at each location, so these need to be combined into one value for a scalar (rather than vector) topographic representation. The simplest way to do this is to take the Root Mean Square (RMS) of the two gradiometers at each location (i.e., estimate the 2D vector length). In SPM, this will create a new sensor type called **MCOMB**. Note that this step is NOT necessary for source reconstruction (where the forward model captures both gradiometers). Note also that the RMS is a non-linear operation, which means that zero-mean additive noise will no longer cancel by averaging across trials, in turn meaning that it is difficult to compare conditions that differ in the number of trials. To take the RMS, select “**Combine Planar**” from the “**SPM –> M/EEG –> Preprocessing menu**”, highlight “**FileName**”, select the “**Dependency**” button, and choose the Artifact-corrected file above. Change the “**copying mode**” to “**Append planar**”. The file produced will be prepended with “**P.**”

#### Average Trials

To average the data across trials, select “**SPM –> M/EEG –> Averaging –> Averaging**”, and again define the input as dependent on the output of the “**Combine Planar**” module above. Keep the remaining options as the default values. (If you like, you could change the type of averaging from “**Standard**” to “**Robust**”. Robust averaging is a more sophisticated version of normal averaging, where each timepoint in each trial is weighted according to how different it is from the median across trials. This can be a nice feature of SPM, which makes averaging more robust to atypical trials, though in fact it does not make much difference for the present data, particularly given the large numbers of trials, and we do not choose it here simply because it takes much longer than conventional averaging.) Once completed, this file will have a prefix of “**m**”.

#### Contrast Conditions

We can also take contrasts of our trial-averaged data, e.g., to create a differential evoked response (ER) between faces and scrambled faces. This is sometimes helpful to see condition effects, and plot their topography. These contrasts are just linear combinations of the original conditions, and so correspond to vectors with 3 elements (for the 3 conditions here). Select “**SPM –> M/EEG –> Averaging –> Contrast over epochs**”, and select the output of averaging above as in the dependent input. You can then select “**New Contrast**” and enter as many contrasts as you like. The resulting output file is prepended with “**w**”. For example, to create an ER that is the difference between faces (averaged across Famous and Unfamiliar) and scrambled faces, enter the vector [0.5 0.5 −1] (assuming conditions are ordered Famous-Unfamiliar-Scrambled; see comment earlier in “**Prepare**” module), and give it a name via the “**New condition**” label. Or to create the differential ER between Famous and Unfamiliar faces, enter the vector [1 −1 0]. Sometimes it is worth repeating the conditions from the previous averaging step by entering, in this case, three contrasts: [1 0 0], [0 1 0], and [0 0 1], for Famous, Unfamiliar and Scrambled conditions, respectively. These will be exactly the same as in the averaged file above, but now we can examine them, as well as the differential responses, within the same file (i.e., same graphics window when we review that file), and so can also delete the previous “**m**” file.

As with the previous pipeline, if you are short of disk space (particularly if you later run all 16 subjects), the outputs produced from the intermediate stages can be deleted using the “**SPM –> M/EEG –> Other –> Delete**” function (see earlier).

##### Save batch and review

**Figure 4:**
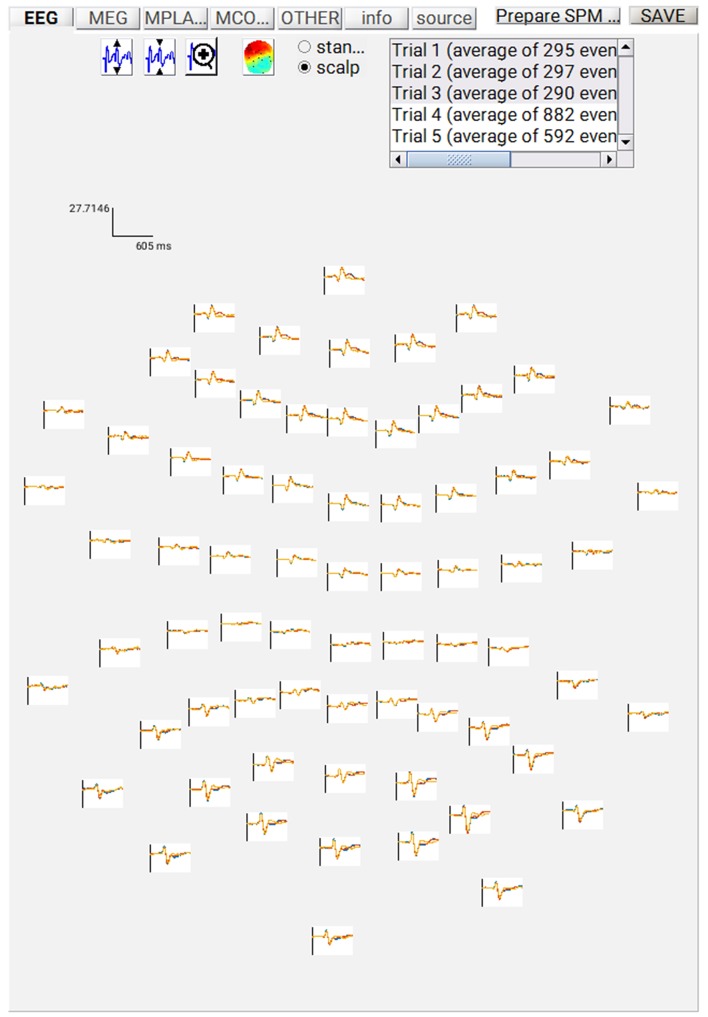
Trial-averaged ERPs for each condition over all EEG channel positions on the scalp.

At this point, you can save the script again. The resulting batch file should look like the “**batch_er_merge_contrast_job.m**” example you downloaded into in the “**code/manual**” directory. We will start by looking at the trial-averaged ERs to each of the three conditions. Select the “**Display**” button on the SPM Menu and select the file “**wmPaMceffdspmeeg_sub-15_ses-meg_task-facere cognition_run-01_proc-sss_meg.mat**”. Then select, for example, the **EEG** tab, and you will see each channel as a row (“strip” or “standard view”) for the mean ER for Famous faces. If you press “**scalp**” instead, the channels will be flat-projected based on their scalp position (nose upwards). You can now display multiple conditions at once by holding the shift-key and selecting Trials 2 and 3 (Unfamiliar and Scrambled) as well. If you press the expand y-axis button (top left) a few times to up-scale the data, you should see something like in Figure 4. You can see the biggest evoked potentials (relative to average over channels) at the back of the head.

**Figure 5:**
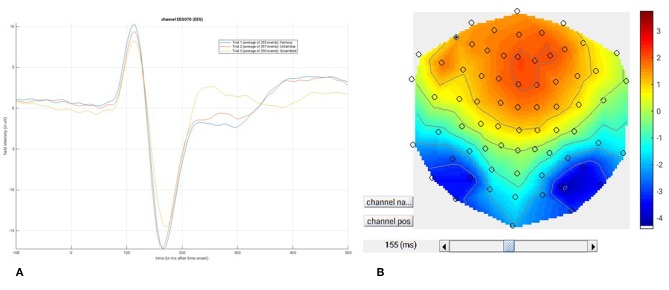
**(A)** Single-channel ERP for each condition, **(B)** Topography for differential ERP for faces minus scrambled faces.

If you press the magnifying glass icon, then with the cross-hairs select Channel 70 (in bottom right quadrant of display), you will get a new figure like in Figure 5A that shows the ERPs for that channel in more detail (and which can be adjusted using the usual MATLAB figure controls). You can see that faces (blue and red lines) show a more negative deflection around 170 ms than do scrambled faces (yellow line), the so-called “N170” component believed to index one of the earliest stages of face processing.

To see the topography of this differential N170 component, select instead the fourth trial (contrast) labeled “Faces—Scrambled”. Then press the colored topography icon, and you will get a new figure with the distribution over the scalp of the face-scrambled difference. If you shift the time-slider on the bottom of that window to the leftmost position, and then repeatedly click on the right arrow, you will see the evolution of the face effect, with no consistent difference during the prestimulus period, or until about 155 ms, at which point a clear dipolar field pattern should emerge (Figure 5B).

You can of course explore the other sensor-types (magnetometers, MEG) and combined gradiometers (**MCOMB**), which will show an analogous “M170”. You can also examine the EOG and ECG channels, which appear under the **OTHER** tab. (Note that the VEOG channel contains a hint of an evoked response: this is not due to eye-movements, but due to the fact that bipolar channels still pick up a bit of brain activity too. The important thing is that there is no obvious EOG artifact associated with the difference between conditions, such as differential blinks).

## Source Reconstruction

### Motivation and Background

The aim of the source reconstruction step is to estimate the distribution of cortical sources that give rise to the MEG/EEG signals observed at the sensor level. This is a non-trivial inverse problem because, for any pattern of sensor values, there could be infinitely many source distributions that would all fit it perfectly (in the same way that infinitely many possible 3D objects produce the same 2D shadow). To arrive at a unique solution, additional constraints (regularization terms or priors) must be introduced, and depending on the nature of these constraints, different solutions could be obtained for the same data. Here we will focus on the “imaging” or “distributed” solution to the inverse problem, specifically two approaches that minimize the L2-norm of the data fit and regularization term(s), either with a uniform prior on the variance of source activities (similar to the classical Minimum-Norm Estimate, MNE), or with multiple, localized regularization terms that encourage a sparse solution (called “MSP” in SPM for “Multiple Sparse Priors”). Note that SPM does offer other inverse solutions, such as a Bayesian implementation of Equivalent Current Dipoles (Kiebel et al., 2008), and also Dynamic Causal Modeling (DCM; David et al., 2006), which can be viewed as a type of inverse solution.

The imaging solution assumes that the sensor-level activity is a result of summation of a large number of dipolar sources distributed over the cortical sheet. These sources have fixed locations and orientations, and the only unknown quantity is their amplitude. The extent to which each sensor sees each source is given by the so-called “lead-field”. This is a vector that can be computed using models rooted in the known physics of electromagnetic fields (more precisely in approximations to Maxwell's equations). These models are called “forward models” because they solve the opposite of the inverse problem—computing the sensor-level signals when the source distribution is known. The forward problem is linear in the source amplitudes meaning that the combined effect of all the sources can be computed by summing their lead-field vectors multiplied by the corresponding source amplitudes. Mathematically this is represented as a matrix multiplication of a lead-field (or gain) matrix L, with the dimensions of number of sensors by number of cortical mesh vertices, and a vector of source current densities J.

Y=LJ+ε 

Y here is the sensor data and ε is random sensor noise. To compute the matrix L it is necessary to provide information about the head geometry, sensor locations and head tissue conductivities. The latter are especially important for EEG. There can be different ways of doing the forward computation which make different simplifying assumptions and achieve different degree of accuracy. In SPM one can use for EEG either a 3-shell spherical model (Cuffin and Cohen, 1979) or a Boundary Element Model (BEM) (Waberski et al., 1998). For MEG there is a choice between a single sphere model, local spheres (Huang et al., 1999) and a single shell model (Nolte, 2003). The latter was shown to perform well in relation to more elaborate BEM models for MEG (Stenroos et al., 2014) and we will use it here. The anatomical information for forward models in SPM can be obtained from the subject's individual structural MRI or from a scaled template head model. Here we will use the former option as we have individual structural images for all the subjects. SPM uses its sophisticated computational neuroanatomy toolkit (Ashburner and Friston, 2005) to obtain individual head and cortical meshes by inverse normalization of template meshes (Mattout et al., 2007). This works much faster than the commonly used FreeSurfer pipeline (Dale et al., 1999) and is robust also for low quality images. An additional advantage is the ability to easily map between individual and canonical anatomy via the use of isomorphic cortical mesh. This ability is important for group inversion and statistical analysis on meshes described below.

The SPM approach to the inverse modeling in the Parametric Empirical Bayes (PEB) framework has been described in several previous publications. Friston et al. (2008) describe the mathematical details of the approach. A more accessible tutorial introduction to the same ideas is given by López et al. (2014). Since the original publication, there have been several extensions of the method, such as introducing group constraints across subjects (Litvak and Friston, 2008), combining different MEG sensor types and EEG in the same inversion (Henson et al., 2009b), using priors derived from fMRI (Henson et al., 2010) and adding a beamforming-like approach to the framework (Belardinelli et al., 2012). A detailed description of the theoretical underpinnings of the analyses shown here is available in Henson et al. (2011).

In brief, it can be shown that assuming that the source activities vector J is sampled from a multivariate normal distribution with zero mean, knowing the covariance matrix of this distribution gives a unique solution for any particular sensor topography. The problem then comes down to estimating this covariance matrix and this is done by representing it as a weighted sum of a relatively small (compared to the number of sources) number of covariance components. Each of these components represents particular assumptions about the source distribution. For instance, an identity matrix component represents the assumption of independent and identically distributed sources that gives a solution equivalent to the classical minimum norm estimate, as noted above (Hämäläinen and Ilmoniemi, 1994). In a similar way it is possible to represent a smoothness constraint similar to that of the Low Resolution Electromagnetic Tomography (LORETA) (Pascual-Marqui et al., 1994). Finally, it is possible to also add components representing activated “patches” on the cortical surface which can be unilateral or bilaterally symmetric. This is the “Multiple Sparse Priors” approach (Friston et al., 2008). Each combination of weights of the covariance components gives a unique inverse solution and can be evaluated in the Bayesian framework by its variational free energy, a cost function combining in a principled way the accuracy (goodness of fit) and complexity of the solution. Computing the inverse solution, therefore, comes down to using a computational optimisation scheme to find the weights of the covariance components that maximize the free energy. There can be different variants of the optimisation scheme and the two currently implemented are called Greedy Search (GS) and Automatic Relevance Determination (ARD). To make the scheme computationally efficient, SPM uses several methods for data reduction and as a consequence it does not work on single topographies but on time windows, and reconstructs the *changes in* activity within the time window rather than activity *per se*. This is different from traditional implementations of inverse solutions used in most other toolboxes, and means that the inverse operator is data-dependent.

### Tutorial Walkthrough

To estimate the cortical sources that give rise to the EEG and MEG data, we will continue to use Subject 15, in order to demonstrate forward and inverse modeling. We need to use the structural MRI of the subject to create a “head model” (that defines the cortex, skull and scalp in terms of meshes) and then a “forward model” (that uses a conductor model to simulate the signal at each sensor predicted by a dipolar source at each point in the cortical mesh).

You can view the structural (T1-weighted) MRI of Subject 15 by displaying the NIfTI file “**sub-15_ses-mri_acq-mprage_T1w.nii**” in the BIDS “**outpth/sub-15/anat**” sub-directory. The approximate position of 3 fiducials within this MRI space—the nasion, and the left and right pre-auricular points—are stored in the file “**sub-15_ses-mri_acq-mprage_T1w.json**” in the same directory (you can type them into SPM's display window when reviewing the MRI to see where they are—note they refer to indices of voxels within the image matrix, not coordinates in real-space). These were identified manually (based on anatomy, and before the face was removed from the MRI images) and are used to define the MRI space relative to the EEG and MEG spaces, which need to be coregistered (see below).

To estimate total power (evoked and induced) of the cortical sources, we need to have the original data for each individual trial. Therefore, our input file will be “**aMceffdspmeeg_sub-15_ses-meg_task-facereco gnition_run-01_proc-sss_meg.mat**” (we could select the trial-averaged file if we just wanted to localize evoked effects). Note that one cannot localize RMS data from combined gradiometers (nor can one localize power or phase data directly).

#### Create Head Model

Select the source reconstruction option in the batch window, and select “**Head model specification**”. Select the file “**aMceffdspmeeg_sub-15_ses-meg_task-facerecognition_run-01_proc-sss_meg.mat**” as the “**M/EEG datasets**”, and the “**inversion index**” as “1” (this index can track different types of forward models and inverse solutions, for example if you want to compare them in terms of log-evidence, e.g., Henson et al., 2009a). Additional comments relating to each index can be inserted if “**comments**” is selected.

The next step is to specify the meshes. Highlight “**meshes**” and select “**mesh source**”. From here select “**Individual structural image**” and select the “**sub-15_ses-mri_acq-mprage_T1w.nii**” file in the BIDS “**outpth/sub-15/anat**” sub-directory. The mesh resolution can be kept as normal (approximately 4,000 vertices per hemisphere). Note that the cortical mesh (and scalp and skull meshes) are created by warping template meshes from a brain in MNI space, based on normalizing this subject's MRI image to that MNI brain (Mattout et al., 2007).

To coregister the MRI and MEEG data, we need to first specify the three fiducials points. You could type each point's 3D coordinates by hand, or more simply, select the option “**Coregistration based on BIDS json file**”, and then select the “**sub-15_ses-mri_acq-mprage_T1w.json**” file in the “**outpth/sub-15/anat**” sub-directory mentioned above. As well as the fiducials, a number of head-points across the scalp were digitized. These were read from the FIF file and stored in the SPM MEEG file. These can help coregistration, by fitting them to the scalp surface mesh (though sometimes they can distort coregistration, e.g., if the end of the nose is digitized, since the nose does not always appear on the scalp mesh, often because it has poor contrast on T1-weighted MRI images, or because face has been removed, as here). If you keep “yes” for the “**use headshape points**” option, these points will be used, but you will notice that alignment of the fiducials is not as good (as if you don't use the headshape points), most likely because the nose points are pulling it too far forward. So here we will say “no” to the “use headshape points” option, so as to rely on the fiducials alone, and trust the anatomical skills of the experimenter. (Alternatively, you could edit the headpoints via the command line or a script so as to remove inappropriate ones).

Finally, for the forward model itself, select EEG head model, and specify this as “**EEG BEM**”; select MEG head model and specify this as “**Single Shell**”. This can then be run. Note that the model parameters are saved, but the gain matrix itself is not estimated until inversion.

##### Save batch and review

**Figure 6:**
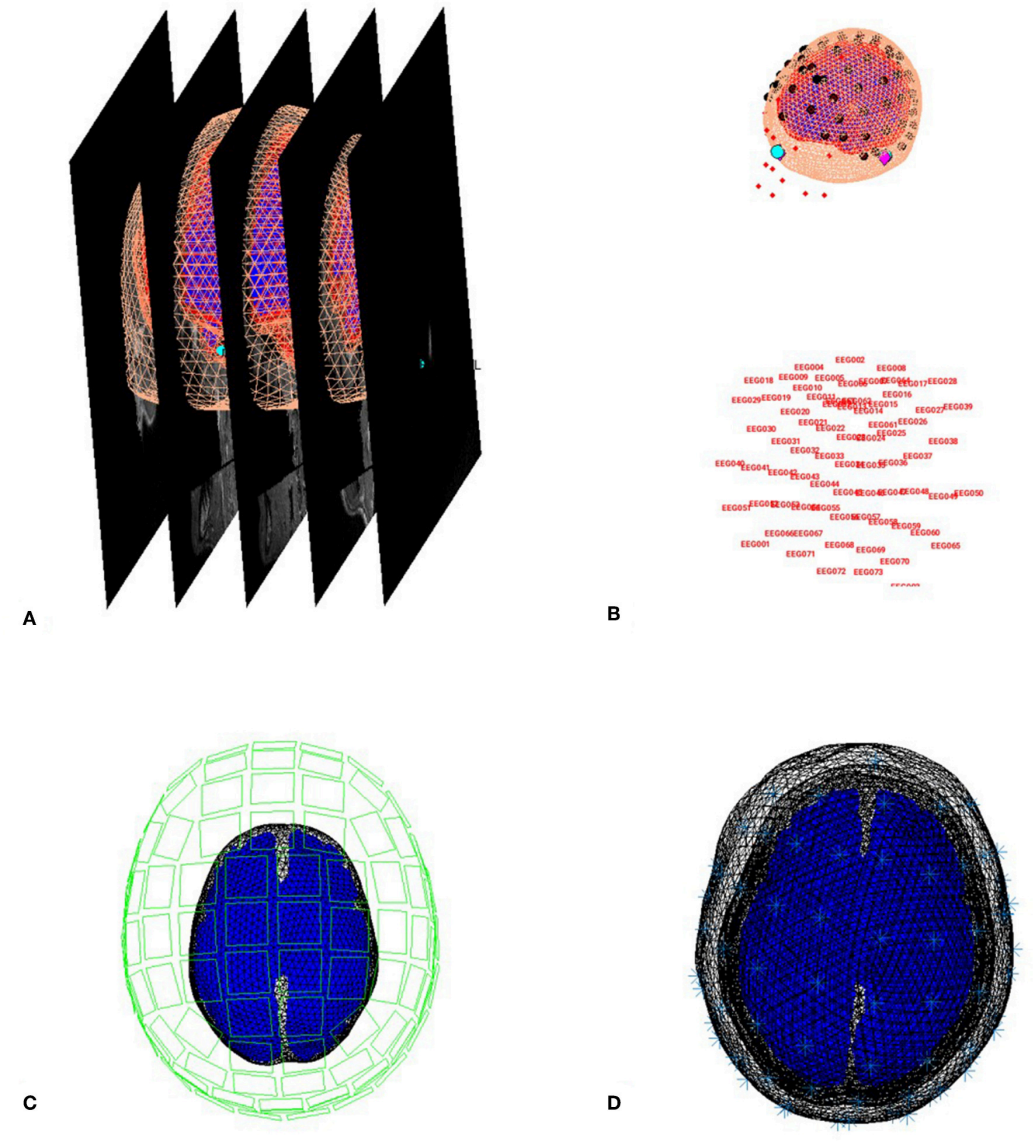
Coregistration of meshes with MRI **(A)** and meshes with EEG and MEG **(B,C)** and surfaces used for forward model **(D)**.

You can now save this inversion batch file (it should look like the “**batch_forward_model_job.m**” file in the “**code/manual**” directory). Once you have run it, you can explore the forward model by pressing the “**3D Source Reconstruction**” button within the SPM Menu window (Figure 1A). This will create a new window (Figure 1C), in which you can select “**Load**” and choose the “**aMceffdspmeeg_ses-meg_task-facerecognition_run-01_proc-sss_meg.mat**” file. On the left hand side of the source localization window, select the “**display**” button below the “**MRI**” button. This will bring up the scalp (orange), inner and outer skull (red) and cortical (blue) meshes of Subject 15's brain, like in Figure 6A (after rotating slightly with MATLAB's 3D tool). Note that the fiducials are shown by cyan disks.

Next, select the “**display**” button beneath “**Co-register**” and then select “EEG” when asked what to display. The graphics window should then display an image like in Figure 6B that displays the electrode locations in black disks, the digitized headpoints in small red dots, the fiducials in the EEG data as purple diamonds, and the MRI fiducials as cyan disks again. The overlap between the EEG fiducials and MRI fiducials indicates how well the data have been coregistered (assuming no error in marking these anatomical features). If you select the “**display**” button beneath “**Forward Model**” and choose EEG or MEG, you should see an image displaying the sensors relative to the surfaces used for the forward model (Figures 6C,D).

#### Model Inversion

We will compare two approaches to inverting the above forward model (both within a Parametric Empirical Bayesian framework). The first one corresponds to a L2-minimum norm, i.e., fitting the data at the same time as minimizing the total energy of the sources. This is called “MNM” (for “Minimum Norm”) or “IID” (for “Independent and Identically-Distributed”) in SPM because it corresponds to assuming that the prior probability of each source being active is independent and identically distributed (i.e., an identity matrix for the prior covariance), but conceptually it is very similar to classical MNE, except that the degree of regularization is estimated as part of the overall model evidence.

Go back to the batch editor, and select “**M/EEG - Source reconstruction – Source Inversion**”. Select the same input file “**aMceffds pmeeg_sub-15_ses-meg_task-facerecognition_ run-01_proc-sss_meg.mat**”, and set the inversion index to 1. Highlight “**what conditions to include**” and select “All”. Next highlight inversion parameters, choose “**custom**” and set the inversion type to “IID”. Then enter the time window of interest as “[−100 500]”. Set the frequency window of interest to “[6 40]”. Select “yes” for the “**PST Hanning window**” but do not select any file for source priors (we will add fMRI priors later). Keep all the remaining parameters at their defaults, including the “**Modalities**” as “All” (which will simultaneously invert, or “fuse,” the data from the EEG, magnetometers and gradiometers; Henson et al., 2009b).

The second type of inversion we will examine is unique to SPM, and is called “Multiple Sparse Priors”, which corresponds to a sparse prior on the sources, namely that only a few are active. Go back to the batch editor, add another “**M/EEG - Source reconstruction – Source Inversion**” module, and select the same input files as before (“**aMceffdspmeeg_sub-15_ses-meg_task-facerecognition_run-01_proc-sss_meg.mat**”), but this time set the inversion index to 2. Set the inversion parameters to “**custom**”, but the inversion type to be “GS”. This is one of several fitting algorithms for optimizing the MSP approach: Greedy Search (GS), Automatic Relevance Detection (ARD) and GS+ARD. We choose GS here because it is quickest and works well for these data. The remaining parameters should be made to match the MNM (IID) inversion above.

#### Time-Frequency Contrasts

Here we are inverting the whole epoch from −100 to +500 ms (and all frequencies), which will produce a timecourse for every single source. If we want to localize an effect within the cortical mesh, we need to summarize these 4D data by averaging power across a particular time-frequency window. To do this, select “**M/EEG - Source reconstruction – Inversion Results**”. Specify the input as dependent on the output of the source inversion, and set the inversion index to 1. Here we will define the time window of interest to “[100 250]” and the frequency window of interest to “[10 20]”, based on the results of the group sensor-level time-frequency analyses in Supplementary Appendix 1. For the contrast type, select “evoked” from the current item window, and the output space as “MNI”. Then replicate this module to produce a second “**inversion results**” module, simply changing the index from 1 to 2 (i.e., to write out the time-frequency contrast for the MNM (IID) as well as MSP (GS) solution).

Now the source power can be written in one of two ways: 1) either as a volumetric NIfTI “Image,” or as 2) a surface-based GIfTI “Mesh”. We will chose “Mesh” here to write out GifTI surfaces, keeping the default cortical smoothing of 8.

##### Save batch and review

**Figure 7:**
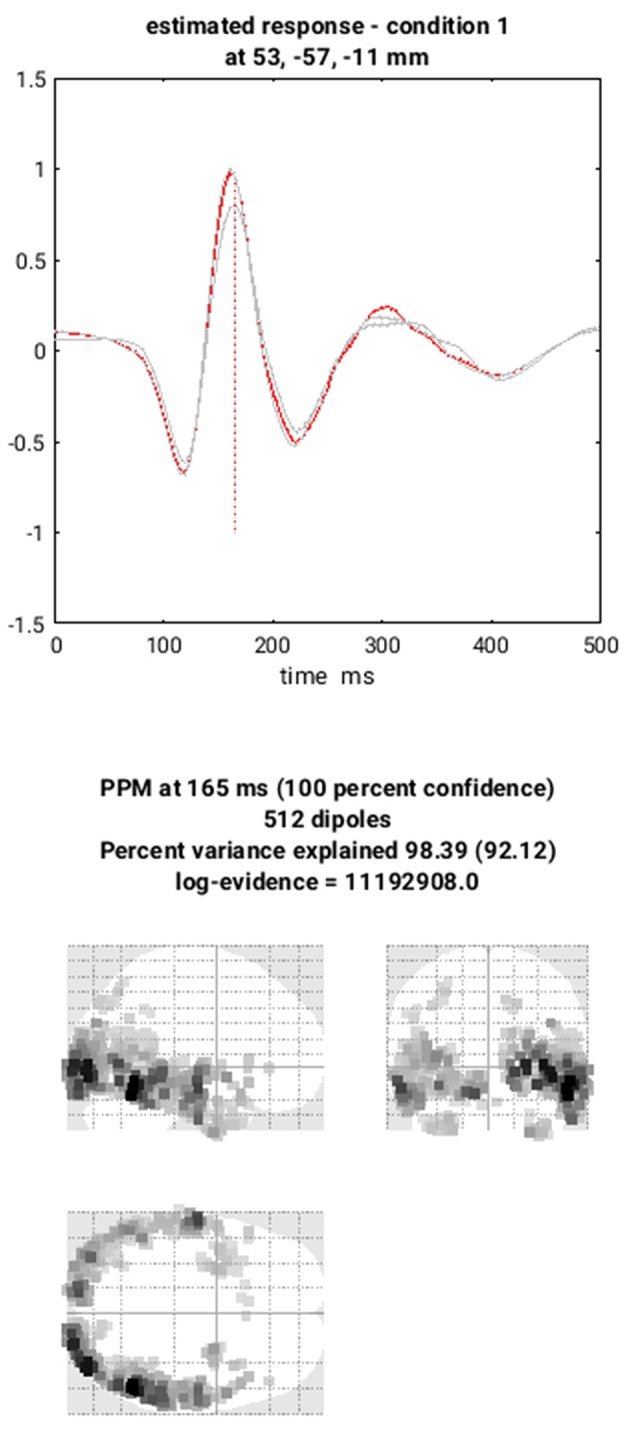
Source solution example MIPs for EEG Modality in subject 15.

You can now save this inversion batch file (it should look like the “**batch_localise_meeg_job.m**” file in “**code/scripted**”). It will take a while to run (because it has to create the gain matrix for the first time), after which you can review the inverse results from within the same “**3D Source Reconstruction**” interface that you used to examine the forward model above. You have to re-“**Load**” the “**aMceffdspmeeg_sub-15_ses-meg_task-facerecognition_run-01_proc-sss_meg.mat**” file. The latest inversion index will be shown (2 in this case), but if you enter 1 for the inversion index, you can see the results of the MNM (IID) inversion. Press the “**mip**” button below the “**Invert**” button, and you should see something like Figure 7. The top plot shows the evoked responses for the three conditions from the peak vertex (at +53 −57 −11, i.e., right fusiform) at 165 ms, with the red line being the currently selected condition, here “1” for Famous faces (press the “condition” button to toggle through the other conditions). If you press “**display**” under the “**Window**” button, you can see a MIP for the time-frequency contrast limited to the 100–250 ms, 10–20 Hz specified above, or if you press the “**display**” under the “**Image**” button, you will see a rendered version.

If you press the “**next**” button to select index 2, you can select the MSP inversion. Press the “**mip**” button again, and you should see results that are sparser and deeper inside the brain, in medial and anterior temporal cortex. One important innovation of SPM's source reconstruction code is the ability to compare different model assumptions (e.g. MNM vs. MSP) in terms of their Bayesian model evidence. In this case, this MSP solution has a higher model evidence, so is more likely to have generated the data. We will compare these two inverse solutions in a different way when we do group statistics below.

If you like, you can also explore other inversion options, either with batch or with this reconstruction window (e.g., creating new inversion indices, though keep in mind that the associated “**aMceffdspmeeg_sub-15_ses-meg_task-facerecognition_run-01_proc-sss_meg.mat**” file can get very large).

##### Create a script for analysis across subjects

Now that we have created a pipeline for forward and inverse modeling, we can script it to run on the remaining 15 subjects. An example is given in the “**batch_master_script.m**” in the “**code/manual**” directory: 
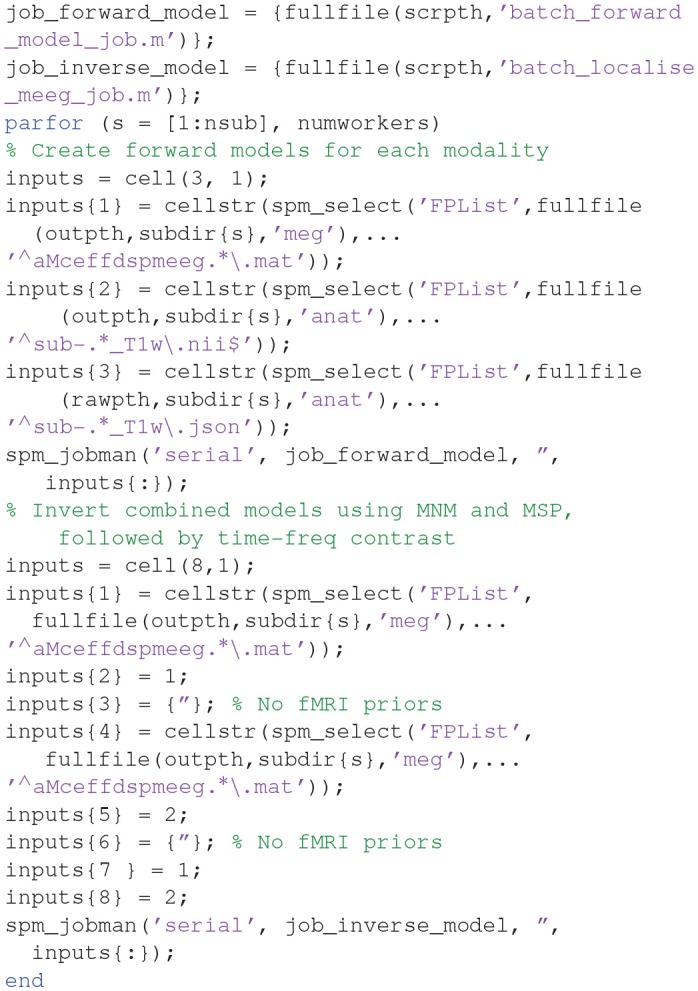


Once you have run this script, we can do statistics on the source power GIfTI images created for each subject.

## Group and fMRI Optimized Source Reconstruction

### Motivation and Background

Because of the indeterminacy of the inverse problem, it is helpful to provide as many constraints as possible. One constraint is to assume that every subject has the same underlying source generators, that are simply seen differently at the sensors owing to different anatomy (head models) and different positions with respect to the sensors (forward models). In the Parametric Empirical Bayes (PEB) framework, this corresponds to assuming the same set of source priors across subjects (allowing for different sensor-level noise; see Litvak and Friston, 2008). This group-based inversion can be implemented in SPM simply by selecting multiple input files to the inversion routine.

A second constraint is to use prior spatial information, i.e., significant clusters from the group fMRI analysis of the same subjects (see Supplementary Appendix 2). This corresponds to an asymmetric integration of multiple modalities, because the significant fMRI clusters are used as priors for the group-optimized source reconstruction of the fused MEG and EEG data (rather than the fMRI data being simultaneously fit in a symmetric integration; see Henson et al., 2011, for further discussion of symmetric vs. asymmetric integration of fMRI and M/EEG). Below, each fMRI cluster will become a separate prior, allowing for the fact that activity in those clusters may occur at different times relative to the time window being localized (which cannot be distinguished by the poor temporal resolution of fMRI). Because SPM's inversion algorithm estimates the weighting (hyperparameter) for each cluster separately, and because there are hyperpriors on those weightings that tend to shrink them to zero, priors that are not helpful in maximizing the variational free energy become discounted, i.e., the fMRI clusters are “soft” priors, allowing a form of “automatic relevance detection”. Henson et al. (2010) confirmed this behavior in practice: when added to a minimum-norm inversion, invalid fMRI priors were generally discounted, but valid priors were kept, and increased the variational free energy (model log-evidence).

### Tutorial Walkthrough

We will use an image of suprathreshold clusters for the contrast of “faces > scrambled” faces from the group fMRI analysis described in Supplementary Appendix 2. This image contains three clusters (left and right occipital face areas and right fusiform face area), each of which will become a separate source prior. This image is stored in the “**spmT_0002_05cor.nii**” file available from the Figshare link provided earlier.

We can combine group-based optimisation of fMRI priors using code like below: 
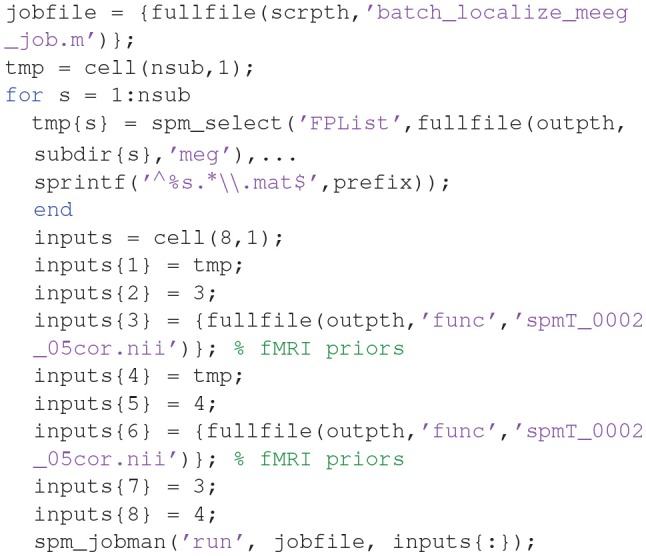


In each subject's “**aMceffdspmeeg_sub-15_ses-meg_ task-facerecognition_run-01_proc-sss_meg.mat**” file, these group-optimized reconstructions using fMRI-priors will be indexed as 3 and 4, so you can compare with the previous MNM and MSP reconstructions indexed as 1 and 2 (by pressing the index button after loading this file via the “**3D Source Reconstruction**” window described above). You can compare the model log-evidences to see which set of constraints is most likely to have generated the sensor data (for a given subject). Below, we will compare group statistics for the four different reconstructions.

## Group Statistics on Source Reconstructions

### Motivation and Background

Neuroimaging data analyses in most cases produce results in the form of signals (e.g., single channel evoked response), 2D images (e.g., time-frequency image of an induced response), 3D volumes (e.g., source reconstruction result) or multidimensional arrays (e.g., a series of source images for adjacent windows in peri-stimulus time). We will refer to all of these as “images” here. When applying statistical analyses to images, it is important to note that in most cases they are inherently smooth e.g., the values for adjacent time points in an evoked response or for adjacent voxels in a functional brain image are correlated across different realizations of the image (e.g., across subjects or trials). A classical statistical test applied to an image will produce an image of the test statistics (e.g., T or F) and a corresponding image of p-values. When using a threshold α for the control of false positive rate (e.g., *p* < 0.05), it is expected that under the null hypothesis (when there is no true effect in the data), for a fraction of tests corresponding to α, we will reject the null hypothesis falsely by chance. Therefore, when each test looks at an image of values with often thousands or more pixels or voxels, it is guaranteed that many of them will be deemed significant unless a correction for multiple comparisons is applied. One way to perform this kind of correction is to control the family-wise error rate (FWER): the probability of rejecting the null hypothesis for any voxel over the whole image. For independent observations, the FWER scales with the number of observations, such that a simple method for controlling FWER is the Bonferroni correction. However, this procedure is rarely adopted in neuroimaging because neighboring observations are often correlated, i.e., for smooth data, Bonferroni correction is too conservative.

SPM uses a different kind of correction based on Random Field Theory (RFT) (Worsley et al., 1992). It is based on mathematical insights into the properties of noise images of certain smoothness. These insights make it possible to quantify the likelihood of an excursion of a certain amplitude in these images. The threshold for an excursion can then be analytically computed to set the probability of crossing it anywhere in the image to α. Excursions exceeding the threshold are treated as significant effects. This approach is often referred to as “peak-level” correction and has been shown to be robust under a wide range of circumstances. RFT can also be applied to predict the probability of excursions based on their spatial extent rather than amplitude (Friston et al., 1994b). This approach is called “cluster-level” correction. It requires defining a “cluster forming threshold” which is an extra parameter in the analysis that alters the sensitivity of the test to large excursions of small amplitude vs. small excursions of large amplitude. Concerns have recently been raised about the cluster-level correction not controlling the FWER at the stated level (Eklund et al., 2016). The underlying issue has to do with the fact that the cluster-level procedure relies on additional assumptions compared to peak-level, and these assumptions only hold for sufficiently high cluster-forming thresholds (Flandin and Friston, 2017). The default uncorrected threshold in SPM of *p* < 0.001 is suitable for cluster-level inference, but popular less conservative thresholds such as *p* < 0.01 and *p* < 0.05 are not.

The statistical parametric mapping approach that gave its name to the SPM toolbox combines RFT with the use of the General Linear Model (GLM). GLM is a generic statistical framework that includes, as particular cases, many commonly used univariate statistical designs, such as dependent and independent samples *t*-tests, Analysis of Variance (ANOVA) and multiple regression. An essential element of the GLM is the design matrix, which is often shown as an image in reports generated by SPM. The rows in this matrix correspond to the images in the test (e.g., for each subject or trial), while the columns represent the independent variables specified by the experimenter to explain the data. These variables can be binary indicators (e.g., whether an image belongs to group A or group B) or real numbers (e.g., age or reaction time). The model is fit to each voxel in the data (this is called a “mass-univariate” approach) and the result is a set of coefficients for each column of the design matrix that minimize the residual not explained by the model in the least squares sense. These coefficients can also be represented as an image of the same type and dimensions as the inputs.

The outputs of GLM fitting can be interrogated by specifying contrasts. T-contrasts test whether any linear combination of column coefficients is either positive or negative. This is useful, for example, to ask whether the signal in group A is higher than in group B. F-contrasts test whether some part of the design (which can be specified as a combination of T-like contrasts or a set of columns in the design matrix) explain significant amount of variance in the data. Each T- or F-contrast generates a corresponding statistical image of T- or F- statistic, respectively, and these are the images to which RFT can be applied to identify significant effects. The GLM can also be applied in a hierarchical fashion using the summary statistic approach. For example, the images of coefficients for linear regression across trials in each subject can be subjected to an independent-samples *t*-test at the between-subject level to compare patients and controls.

A detailed description of the GLM is outside the scope of the present paper and the interested reader is referred to the original paper that introduced GLM to neuroimaging (Friston et al., 1994a) as well as the more recent discussion of the application of this approach to MEG/EEG (Kilner and Friston, 2010).

### Tutorial Walkthrough

#### Model Specification

Open a new batch, select “**Factorial design specification**” under “**Stats**” on the “**SPM**” toolbar at the top of the batch editor window. We will show here how to set up a statistical analysis on meshes for the individual MNM inversions. The other analyses are analogous and will be set-up automatically with a script.

The first thing is to specify the output directory where the SPM stats files will be saved. So first create such a directory “**outpth/meg/IndMNMStats**”. Highlight “**Design**” and from the current item window, select “**One-way ANOVA–within subject**” (somewhat confusingly, this is not an analysis within one subject, but an analysis in which multiple measures come from “within” each subject, also called a “repeated-measures ANOVA”). Highlight “**Subjects**” and create a “**New:subject**”. In the “**scans**” field, you can now select 3 source power GIfTI images for the first subject that have been created in the “**sub-01/meg/**” folder and enter the “**Conditions**” as “[1 2 3]”. It is important for the contrasts below that you select the files in the order Famous-Unfamiliar-Scrambled. You can then select “**Replicate: Subject**” under the “**Subjects**” item, keeping the “**Conditions**” unchanged, but changing the “**Scans**” to those in “**sub-02/meg/**”. You can then repeat these steps for the remaining subjects. Or if you prefer (as it is cumbersome with the GUI), you can create 16 blank “**Subject**” items, save the batch script, and then populate the “**Scans**” field (and “**Conditions**” field) via a MATLAB script. Finally, set the “**Variance**” to “Unequal” and the “**Independence**” to “No” (to model the error correlation, i.e., non-sphericity, Friston et al., 2002). Keep all the remaining defaults.

This now completes the GLM specification, but before running it, we will add two more modules.

#### Model Estimation

The next step within this pipeline is to estimate the above model. Add a module for “**Model estimation**” from the “**Stats**” option on the SPM toolbar and define the file name as being dependent on the results of the factorial design specification output. Leave the other options with their default value.

#### Setting Up Contrasts

The final step is to add a module for creating contrasts from “**SPM->Stats->Contrast Manager**”. Define the file name as dependent on the model estimation. The first contrast will be a generic one that tests whether significant variance is captured by the first 3 regressors. This corresponds to an F-contrast based on a 3 × 3 identity matrix. Highlight “**Contrast sessions**” and select a “**new F-contrast**”, using the current item module. Name this contrast “All Effects”. Then define the weights matrix by typing in “eye(3) ones(3,16)/16” (which is MATLAB for a 3 × 3 identity matrix, followed by 1/16 for each of the 16 subject effects; the latter being necessary if one wants to see absolute changes in power vs. baseline). You can use this contrast to plot the parameter estimates for the 3 conditions for a given voxel, if you want.

More interestingly perhaps, we can also define a contrast that compares faces against scrambled faces. So this time make a T-contrast, name this one “**Faces (Fam+ Unf)> Scrambled**”, and type in the weights **“[0.5 0.5–1]**”. (If you want to look at power decreases, you can create another T-contrast and reverse the sign of these contrast weights).

##### Save batch and review

Once you have added all the contrasts you want, you can save this batch file (it should look like the “**batch_stats_rmANOVA_job.m**” file in the **code/manual** directory, though that example also includes some additional contrasts that might be of interest, but which we have not created here).

Now we want to repeat this ANOVA on the remaining three inversions, i.e. four in total, crossing MSP vs. MNM inversion, with group inversion with vs. without fMRI priors. We can script this, like below: 
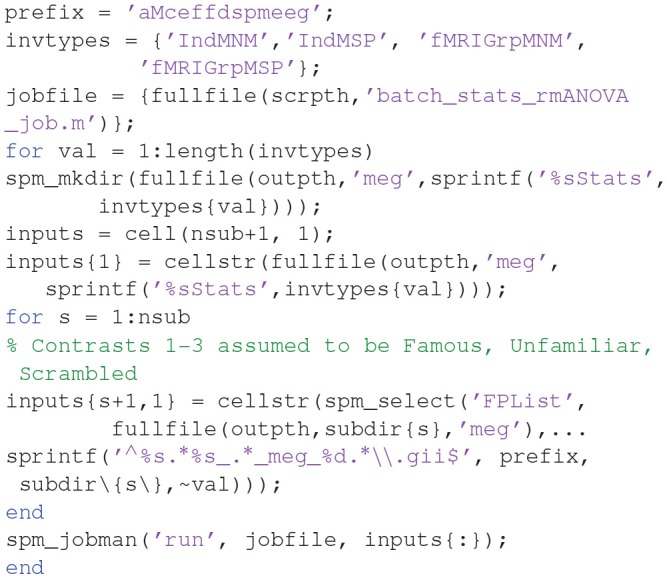


(where the “Ind” prefix in the output directories refers to “individual” source reconstructions and “fMRIGrp” to group-optimized inversions with fMRI priors).

##### Viewing results

**Figure 8:**
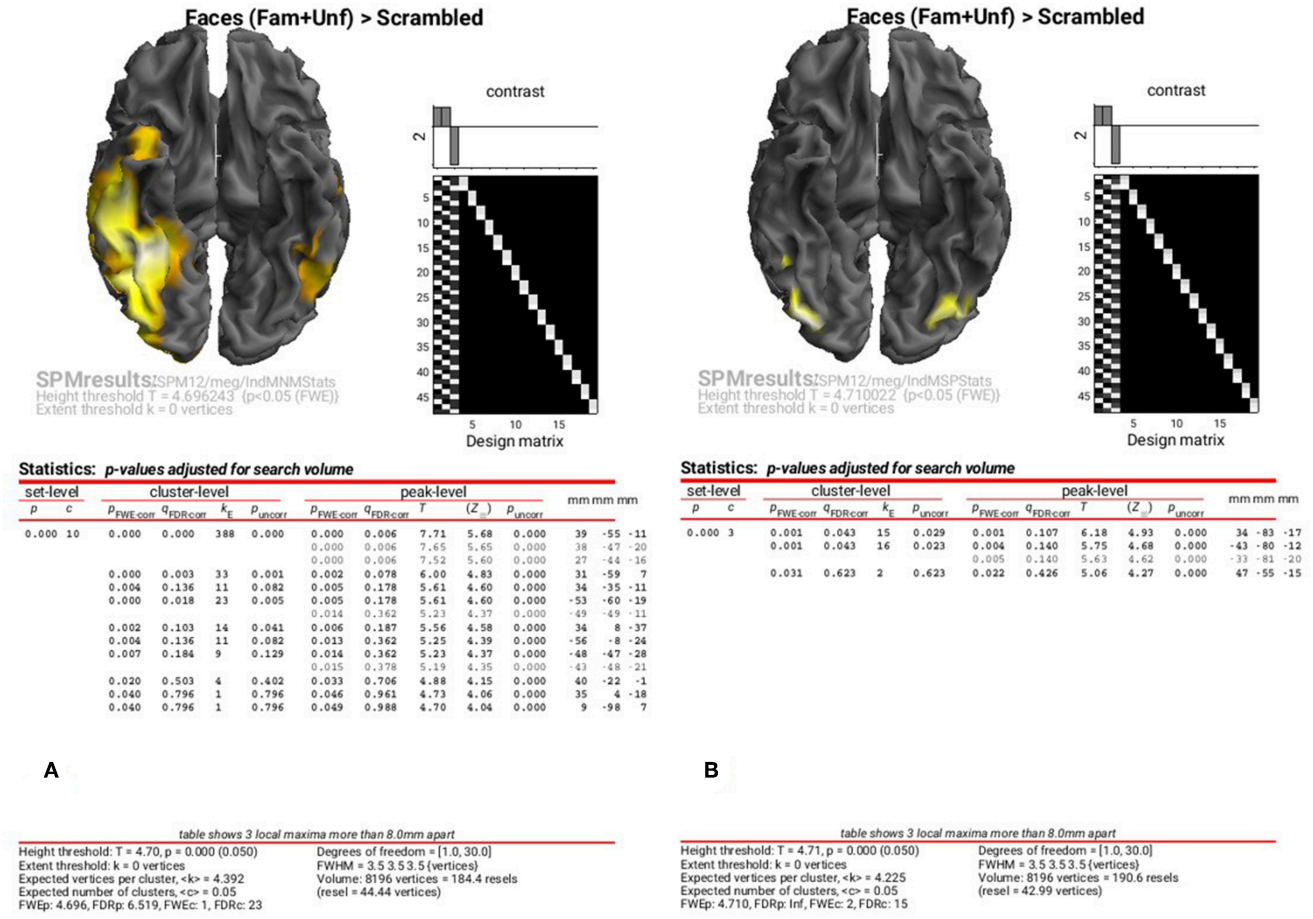
Group SPM for Faces vs. Scrambled power on cortical mesh between 10 and 20 Hz and 100 and 250 ms across all 16 subjects at *p* < 0.05 FWE corrected for MNM **(A)** and MSP **(B)**.

The results of the above ANOVAs (GLMs) can be viewed by selecting “**Results**” from the SPM Menu window. Start by selecting the “**SPM.mat**” file in the “**STStats/meg/IndMNMStats**” directory, and from the new Contrast Manager window, select the pre-specified contrast “**Faces (Fam+Unf)>Scrambled**”. Within the “**Stats: Results**” bar window, which will appear on the left hand side, select the following: “**Apply Maskings**” “None”, “**P value adjustment to control**” “FWE”, keep the threshold at 0.05, “**extent threshold {voxels}**” 0; “**Data Type**” “Volumetric 2D/3D”. The top of the Graphics window should then show the maximal intensity projection (MIP) of the suprathreshold voxels, as in Figure 8A (after having right-clicked on the rendered mesh, selecting “**View**” and then “**x-y view (bottom)**”, in order to reveal the underside of the cortex). Note the broad right fusiform cluster, with additional clusters on left and more anteriorly on right. You can compare this to the fMRI group results in Supplementary Figure A2.1, which are similar, but much more focal.

You can also look at the results of the MSP inversion by selecting the “**SPM.mat**” file in the “**MEEG/IndMSPStats**” directory (Figure 8B). This reveals much more focal clusters in right fusiform face area (FFA) and left and right occipital face area (OFA), more like the fMRI (see Supplementary Figure A2.1).

**Figure 9:**
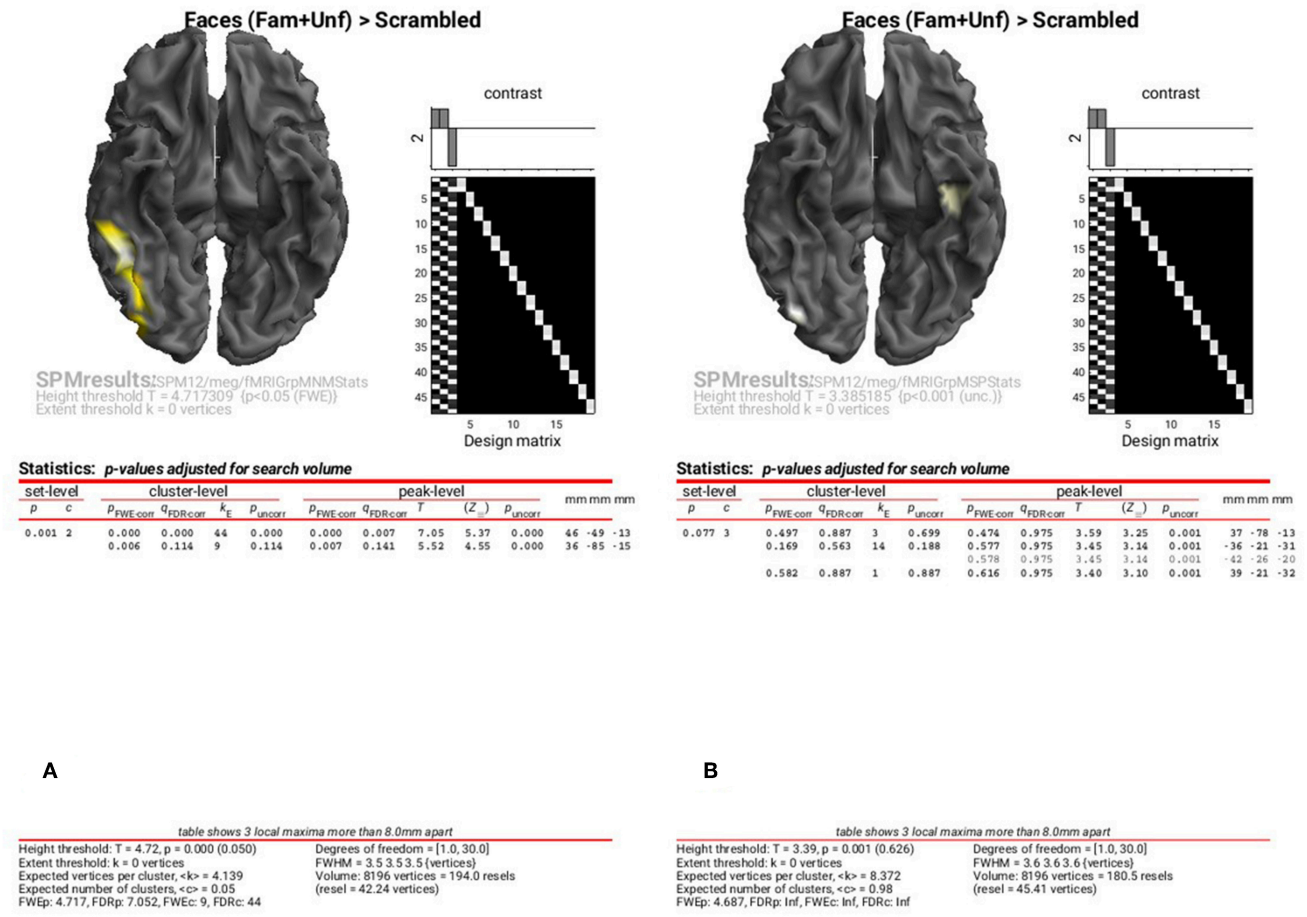
Group SPM for Faces vs. Scrambled power on cortical mesh between 10 and 20 Hz and 100 and 250 ms across all 16 subjects using group-optimisation of MNM **(A)** and MSP **(B)** together with fMRI priors. **(A)** is thresholded at *p* < 0.05 FWE corrected, **(B)** at *p* < 0.001 uncorrected.

To examine the results of inversions with group optimisation and fMRI priors, select the “**SPM.mat**” files from “**fMRIGrpMNMStats**” and “**fMRIGrpMSPStats**”, and choose the same corrected threshold. You should see results like in Figure 9A, where the group-optimized fMRI priors have focused the suprathreshold clusters to the right FFA and OFA (cf. Figure 8A). For the MSP inversion however, the addition of fMRI priors does not help much, with nothing surviving a corrected threshold. Lowering the threshold to *p* < 0.001 uncorrected reveals a right OFA and left anterior temporal region (Figure 9B). For further, more formal comparisons of fMRI priors (see Henson et al., 2010).

This concludes this demonstration of SPM12 multimodal integration of MEG, EEG and fMRI, but feel free to explore yet further options in the software (in conjunction with Litvak et al., 2011).

## Author Contributions

RH provided the data, designed, and performed the analysis, and wrote the paper. HA tested the analysis and helped write the paper. GF helped format the data, helped with the SPM software, checked the analysis, and helped write the paper. VL is lead developer of the SPM software for M/EEG, helped with the analysis, and helped write the paper.

### Conflict of Interest Statement

The authors declare that the research was conducted in the absence of any commercial or financial relationships that could be construed as a potential conflict of interest. The handling editor is currently co-organizing a Research Topic with one of the authors VL, and confirms the absence of any other collaboration.

## Footnotes

^1^Note that these results are from a subset of the analyses reported in Chapter 42 of the SPM12 manual, which is available online at https://www.fil.ion.ucl.ac.uk/spm/doc/manual.pdf#Chap:data:multi, but has not previously been published in a peer-reviewed journal.

^2^A non-BIDS version of the same dataset can be found here: ftp://ftp.mrc-cbu.cam.ac.uk/personal/rik.henson/wakemandg_hensonrn.

^3^If the download from a browser fails due to the size of the file, you can use the AWS Command Line Interface (https://aws.amazon.com/cli) with: aws s3 synch –no-sign-request s3://openneuro.org/ds000117/yourpath.

^4^Detailed instructions for installation are available at https://www.wikibooks.org/wiki/SPM#Installation.

^5^In MATLAB, a bandpass filter is not equivalent to the combination of the corresponding high- and low-pass filters. A bandpass filter is more suitable for narrow bands, and bandpass filters with the lower edge very close to zero, such as [0.1 30], can become unstable. When doing low- and high- pass filtering separately it is recommended to start with the high-pass because it removes the DC offset that could create ringing artifacts at the data edges. This is particularly important for filtering epoched data but depending on the magnitude of the DC offset, could also be relevant for continuous data.

^6^The channel definitions and bad channels do not actually depend on run in this dataset, so could be left as is. However, in general they might (e.g., if channels did go bad between runs).
