# Supplementary material for: Multimodal Integration of M/EEG and f/MRI Data in SPM12
Source: Front Neurosci. 2019 Apr 24;13:300. doi: 10.3389/fnins.2019.00300 (PMC6491835; doi:10.3389/fnins.2019.00300)
Supplement: Supplementary file 1 [file Data_Sheet_1.pdf]

Supplementary Material for

Multimodal integration of M/EEG and f/MRI data in SPM12

Richard N Henson[1*], Hunar Abdulraham[1], Guillaume Flandin[2], Vladimir Litvak[2]

[1]MRC Cognition & Brain Sciences Unit, University of Cambridge, Cambridge, UK

[2] Wellcome Centre for Human Neuroimaging, University College London, London, UK

*** Correspondence:**
Corresponding Author
rik.henson@mrc-cbu.cam.ac.uk

Keywords: MEG, EEG, fMRI, multimodal, fusion, SPM, inversion, faces

Appendix 1. Time-Frequency analysis and statistics across subjects

In the main paper, we describe a typical evoked response (ER) analysis, where we simply average across trials in each condition. However this will attenuate any non-phase-locked, i.e. induced responses; to detect these, here we describe an alternative time-frequency (TF) analysis that is performed before averaging.

More specifically, we will use Morlet wavelets to decompose each trial into power and phase across peristimulus time and frequency, for a single channel of each channel-type (i.e., one EEG electrode, on magnetometer and one gradiometer). We will then average the power estimate across trials and create a 2D time-frequency image, which we can then test for reliability across subjects. This assumes that you have already run the script in the main paper that processes all subjects (in fact, here we only need the data after downsampling).

A.1.1 Wavelet Estimation

The first module to be added to the pipeline is the "**Time-Frequency Analysis**" under "**SPM – M/EEG - Time-frequency**". Highlight "**File Name**", choose "**Specify**" box, and select the file "`dspmeeg_sub-15_ses-meg_task-facerecognition_run-1_proc-sss_meg.mat`". This is the continuous (non-epoched) downsampled file from the Downsample section of the main paper.

To save disk space and computation time, we will not run time-frequency analysis over all channels (though you could); instead we will select one channel from each sensor-type. So from "**Channel selection**", delete "**All**" and from the Current Item window press "**New: Custom channel**" 3 times and click "**Specify**" on each one and enter: EEG070, MEG2121, and MEG2543 for an EEG, Magnetometer and Gradiometer channel respectively. Then highlight the "**Frequencies of interest**", click "**Specify**" and enter "6:40" (which will estimate frequencies from 6 to 40Hz in steps of 1Hz). Then highlight "**Spectral estimation**" and select "Morlet wavelet transform". Then highlight "**Number of wavelet cycles**" and specify this as "5". The fixed time window length should be set at 0 as default. To reduce the size of the file further, we will also subsample in time by a factor of 5 (i.e., every 25ms, given the additional downsampling to 5ms done earlier in preprocessing). Do this by selecting "**subsample**" then click "Specify" and enter 5. Finally, select "Yes" for "**Save phase**". This will produce two files, one for power (prefixed with "**`tf`**") and one for phase (prefixed with "**`tph`**").

A.1.2 Epoch

Similar to the Encoding section in the main paper, we need to epoch the data, but now for two files: power and phase. Start by adding one "**Epoching**" module from "**SPM->M/EEG->Preprocessing**". Then use Dependency to select the first output from the time-frequency module, ie for the "**power**" dataset. We then need to define which trials to epoch, exactly the same as in the main paper. So press "**Define Trial**" and enter [-100 500] for the time window. Then on "**Trial definitions**", select "**New:Trial**" and enter "Famous" as the "**Condition label**", "BIDS" as the "**Event type**" and " 'Famous' " as the "**Event value**" (again note the single quotes around the Event value). Then replicate this trial definition twice, and for the second Trial, change the "**Condition label**" to "Unfamiliar" and "**Event**

values" to " 'Unfamiliar' ", and for the third Trial, change the "**Condition label**" to "Scrambled" and the "**Event values**" to " 'Scrambled' ". For the "**Baseline correction**" option, select "No".

You can the right-mouse the "**Epoching**" module and select "replicate". This will produce a second module, with same trial definitions, so you don't have to repeat steps above. All you need to do is change the input file dependency from the "**power**" dataset to the "**phase**" dataset, and then the phase data will be epoched in the same manner.

As in the main paper, you can save the batch and script, then create a loop over all the 6 runs. The resulting batch script should be similar to "`batch_tf_wavelet_epoch_job.m`" in the code/manual directory. The output from these steps are two files: "`etf_dspmeeg_sub-15_ses-meg_task-facerecognition_run-%02d_proc-sss_meg.mat`" and "`etph_dspmeeg_sub-15_ses-meg_task-facerecognition_run-%02d_proc-sss_meg.mat`" for each run.

A.1.3 Merge

This step is the same as in the Merge (Concatenate Runs) section of the main paper, except we need to add the "**Merging**" module twice, since the inputs are now both power and phase from the stage above.

A.1.4 Sort Conditions

As in the main paper, we add a "**Prepare**" Module to sort the conditions before averaging, and we can use "Dependency" to use output of Merging in the previous step as the input of this module. Add this module twice for both power ("`tf`") and phase ("`tph`") files.

A.1.5 Average

We now need to add two "**Averaging**" modules, one with Dependency on the power file from the first Prepare step, and the other with Dependency on the phase file from the second Prepare step. For the power averaging, choose straight averaging and keep the other values as default. For the phase averaging however, we need to additionally select "Yes" to the "**Compute phase-locking value**". This corresponds to "circular" averaging, because phase is an imaginary number. This produces a scalar quantity called the phase-locking value (PLV). The prefix will be "`m`" in both cases.

A.1.6 Baseline rescale

It often helps to express power as proportional changes relative to a baseline period (this also helps normalise power changes across frequencies, since power at higher frequencies tends to be much lower than power at lower frequencies). If you only care about the average, then this normalisation (rescaling) is best done after averaging, because the divisive normalisation of single trials amplifies noise (Ciuparu & Mureşan, 2016). To implement this rescaling, we need to select the "**Time-Frequency Rescale**" option from the "**Time-Frequency**" menu (note: not the "Baseline Correction" module from the "Preprocessing" menu). There are several options available. For the power, which is a rectified, second-order measure, it also helps to apply a log transform. We will therefore use the log-ratio ("**LogR**") option, where all power values at a given frequency are divided by the mean power from -100 to 0ms at that frequency, and the log taken (which is equivalent to subtracting the

log of the baseline power). Select the power file output from the previous phase as the dependency, select the log ratio option, and enter the baseline time window as [-100 0]. The output file prefix will be "`r`". We will not bother to baseline-correct the phase-data.

A.1.7 Contrast conditions

Finally, as in the main paper, we can take contrasts of our trial-averaged data, e.g., to create a time-frequency image of the difference in power, or in PLV, between faces and scrambled faces. Create two "**SPM –> M/EEG –> Average –> Contrast over epochs**" modules, one with the average power file as input, and one with the averaged phase (PLV) file as input. You can then select "**New Contrast**" and enter contrasts like [0.5 0.5 -1] (for faces vs scrambled; see earlier) and [1 -1 0] (for familiar vs unfamiliar). The resulting output file is prepended with "`w`".

As before, if you want to save file-space, you can add further "**Delete**" modules, since we will not need many of the intermediate files. The only files we need to keep are the averaged power and phase files (since these are used to create time-frequency images below) and the contrasted versions (for visual inspection of effects within each subject).

A.1.8 Create 2D time-frequency images

Below we will repeat the above steps for every subject, and create a statistical parametric map of power and PLV changes across subjects, in order to localise face-induced power and phase-locking changes in frequency and time. This requires that we create 2D images, one per condition (per subject). Select the "**convert2images**" option and select the baseline-rescaled, trial-averaged power file as the dependency from the stage above. Select the mode "**time x frequency**". Next select channel selection by type. Here, choose EEG for "**Channel selection**" and define the prefix as "`eeg_img_pow`". (Of course, this module can be repeated for MEG and MEGPLANAR sensors if wanted.)

Save batch and review

You can now save this time-frequency batch file (it should look like the "`batch_tf_merge_contrast_job.m`" file in the "`code/manual`" directory). Once you have run it, you can then review the contrast files, e.g, for power ("`wrmcetf_dspmeeg_sub-15_ses-meg_task-facerecognition_run-01_proc-sss_meg.mat`") or PLV ("`wrmcetph_dspmeeg_sub-15_ses-meg_task-facerecognition_run-01_proc-sss_meg.mat`"). When displaying the files, you will now see only one channel per sensor-type; for the EEG data, when you select the trial-type corresponding to the contrast of faces versus scrambled faces, you should see something like Figure A.1.1A for power, and Figure A.1.1B for PLV.

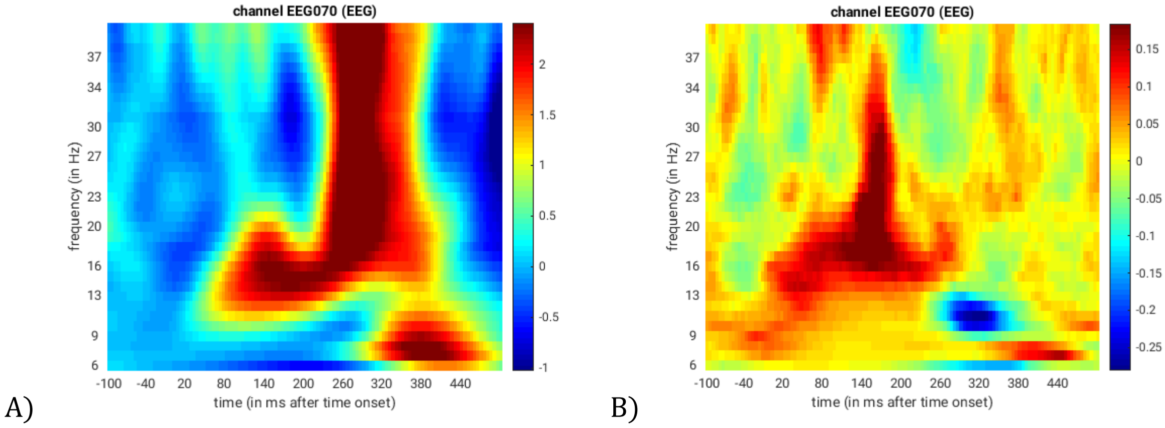

Figure A1.1 A) Trial-averaged power for faces > scrambled in EEG channel 70 (right posterior). B) Trial-averaged PLV for faces > scrambled in EEG channel 70 (right posterior).

Creating a script for analysis across subjects

Now that we have created a pipeline for the time-frequency analysis (consisting of two job files, each job file containing a handful of modules), we want to run this on all subjects. First you need to clear all the values of the input files to the `batch_tf_wavelet_epoch_job.m` and `batch_tf_merge_contrast_job.m`, and re-save them as job files (so that we can pass input files to them that depend on the subject). For this, a bit of MATLAB knowledge is required to call these pipelines within a "`for`" (or "`parfor`") loop across subjects, e.g:

```
parfor (s = [1:nsub], numworkers)
    if numworkers > 0
        spm_jobman('initcfg');
        spm('defaults', 'EEG');
        spm_get_defaults('cmdline',true);
    end

    % Change to subject's directory
    swd = fullfile(outpth,subdir{s},'meg');
    cd(swd);

    runs = spm_BIDS(BIDS,'runs', 'sub',subs{s}, 'modality','meg',...
        'type','meg');
    nrun = numel(runs);

    jobs_tf_wavelet = repmat({fullfile(scrpth,'batch_tf_wavelet_epoch_job.m')}, 1, nrun);
    jobs_tf_merge =          {fullfile(scrpth,'batch_tf_merge_contrast_job.m')};

    % Wavelets and epoching
    inputs = cell(1, nrun);
    for r = 1:nrun
        inputs{1, r} = cellstr(fullfile(swd,...
            sprintf('dspmeeg_sub-%02d_ses-meg_task-facerecognition_run-%02d_proc-
sss_meg.mat',s,r)));
    end
    spm_jobman('serial', jobs_tf_wavelet,'', inputs{:});

    % Merge to contrast
    inputs  = cell(2, 1);
    inputs{1} = cellstr(spm_select('FPList',fullfile(swd),'etf_dspmeeg.*\.mat$'));
    inputs{2} = cellstr(spm_select('FPList',fullfile(swd),'etph_dspmeeg.*\.mat$'));

    spm_jobman('serial', jobs_tf_merge, '', inputs{:});
end
```

A.1.9. Time-frequency Stats across Subjects

We can now enter the frequency-time power images (one per condition per subject) into a group (2nd-level) analysis that corresponds to repeated-measures ANOVA, like for the source-space SPM analysis in the main paper.

The first thing is to specify the output directory where the SPM stats files will be saved. Because this is now an analysis across subjects, we can create a directory above "**`sub-15`**" in the directory tree. So create a new top-level directory called, for example, "**`MEEG`**" (because in the main paper we have group stats source-reconstructed MEEG data too), then a sub-directory within it called "**`TFStats`**", then two sub-directories called "**`PowStats`**" and "**`PLVStats`**", each with a further sub-directory called "**`eeg`**" (and potentially two more called "**`mag`**" and "**`grd`**" if you want to examine other sensor-types too).

A.1.10 Model Specification, Estimation and Contrasts

To create this repeated-measures ANOVA, open a new batch and select "**Factorial design specification**" under "**Stats**" on the "**SPM**" toolbar. Then select this new "**`MEEG/TFStats/PowStats/eeg`**" directory (for the first, power analysis).

Highlight "**Design**" and from the current item window, select "**One-way ANOVA–within subject**". Highlight "**Subjects**" and create a "**New: subject**". In the "**scans**" field, you can now select the 3 power images for the first subject (which should have been created in the "`sub-01/meg/eeg_img_pow_rmcetf_dspmeeg_sub-01_ses-meg_task-facerecognition_run-01_proc-sss_meg`" directory if you ran the script above), and enter the "**Conditions**" as "[1 2 3]". It is important for the contrasts below that you select the files in the order Famous-Unfamiliar-Scrambled. You can then select "**Replicate: Subject**" under the "**Subjects**" item, keeping the "**Conditions**" unchanged, but changing the "**Scans**" to those in "`sub-02/meg/eeg_img_pow_rmcetf_dspmeeg_sub-01_ses-meg_task-facerecognition_run-01_proc-sss_meg`". You can then repeat these steps for the remaining subjects. Or if you prefer (because this is a pain to do via the GUI!), you can create 16 blank "**Subject**" items, save the batch script, and then populate the "**Scans**" field (and Conditions field) via a MATLAB script. Finally, set the "Variance" to "Unequal" and the "Independence" to "No" (to model the error correlation, i.e., nonsphericity, mentioned above). Keep all the remaining defaults.

The next step is to add a module for "**Model Estimation**" from the "**Stats**" option and define the file name as being dependent on the results of the factorial design specification output. Keep the remaining defaults.

The final step is to add a module for "**Creating contrasts**". Define the file name as dependent on the model estimation. The first contrast will be a generic one that tests whether significant variance is captured by the first 3 regressors. This corresponds to an F-contrast based on a 3x3 identity matrix. Highlight contrast sessions and select a new F- contrast session, using the current item module. Name this contrast "All Effects". Then define the weights matrix by typing in "eye(3) ones(3,16)/16"

(which is MATLAB for a 3x3 identity matrix, followed by 1/16 for each of the 16 subject effects; the latter being necessary if one wants to see absolute changes in power vs baseline). You can use this contrast later to plot the parameter estimates for the 3 conditions.

More interestingly perhaps, we can also define a contrast that compares faces against scrambled faces, i.e., to test whether the average power increase across trials seen in Channel 70 of Subject 15 in Figure A1.1 is reliable when averaging across channels and across subjects. So this time make a T-contrast, name this one "Faces (Fam+ Unf) > Scrambled", and type in the weights "0.5 0.5 -1". (If you want to look at power decreases, you can create another T-contrast and reverse the sign of these contrast weights.)

Save batch and review

Once you had added all the contrasts you want, you can save this batch file (it should look like the "`batch_stats_rmANOVA_job.m`" file in the `code/manual` directory). Then run it, and when it has finished, press "**Results**" from the SPM Menu window. Select the "`SPM.mat`" file in the "`MEEG/TFStats/eeg`" directory, and from the new Contrast Manager window, select the pre-specified T-contrast "Faces (Fam+Unf) >Scrambled". Within the "Stats: Results" bar window, when given the option, select the following: Apply Masking: None, P value adjustment to control: none, keep the threshold at 0.001, extent threshold {voxels}: 0; Data Type: Time-frequency. The Graphics window should then show what is in Figure A1.2 below. Note the peak increase in power at 12Hz, 165ms that survives correction for its spatial extent, which most likely corresponds to the N170. We can use the approximate extent of this cluster to define the time-frequency contrast for the source reconstruction described in the main text. There is also a second cluster slightly later cluster at much higher frequencies (~28Hz) which may be largely induced rather than evoked, and not readily apparent the evoked analysis of the main paper.

You can also repeat the above SPM statistical analysis on the PLV data for each subject. If you do this for the EEG channel, you should see results like those in Figure A1.3, which show an increase in phase-locking around a similar time and frequency as the above power changes. This distinction between stimulus-locked changes in power versus phase is not possible with our previous evoked response analysis, and suggests that faces cause a change in phase of ongoing (alpha) oscillations, as well as a modulation of their power.

Faces (Fam+Unf) > Scrambled

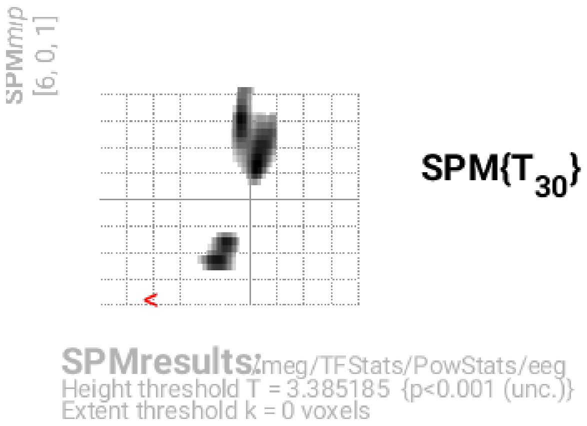

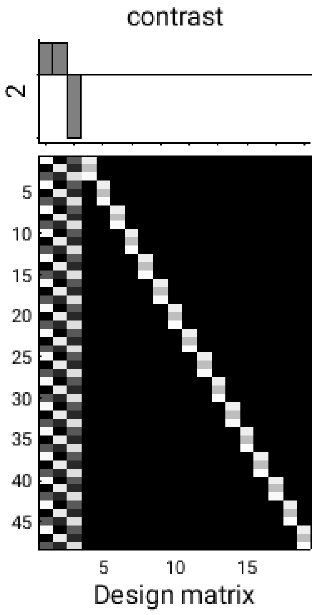

Statistics: ***p-values adjusted for search volume***

set-level		cluster-level				peak-level					Hz ms	
p	c	$p_{FWE-corr}$	$q_{FDR-corr}$	k_E	p_{uncorr}	$p_{FWE-corr}$	$q_{FDR-corr}$	T	$(Z_{\equiv})$	p_{uncorr}		
0.030	2	0.000	0.000	227	0.000	0.063	0.294	4.02	3.57	0.000	28 255	1
						0.068	0.294	3.99	3.54	0.000	35 220	1
		0.003	0.012	77	0.012	0.075	0.294	3.95	3.51	0.000	12 165	1
						0.076	0.294	3.94	3.51	0.000	15 185	1

table shows 3 local maxima more than 8.0mm apart

Height threshold: T = 3.39, p = 0.001 (0.236)
Extent threshold: k = 0 voxels
Expected voxels per cluster, <k> = 17.441
Expected number of clusters, <c> = 0.27
FWEp: 4.128, FDRp: Inf, FWEc: 77, FDRc: 77

Degrees of freedom = [1.0, 30.0]
FWHM = 7.2 65.5 Hz ms ; 7.2 13.1 {voxels}
Volume: 21175 = 4235 voxels = 43.1 resels
Voxel size: 1.0 5.0 Hz ms ; (resel = 94.59 voxels)

Faces (Fam+Unf) > Scrambled

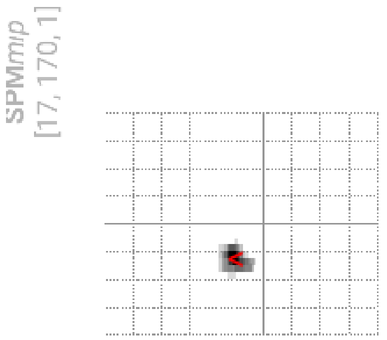

SPM{T_{30}}

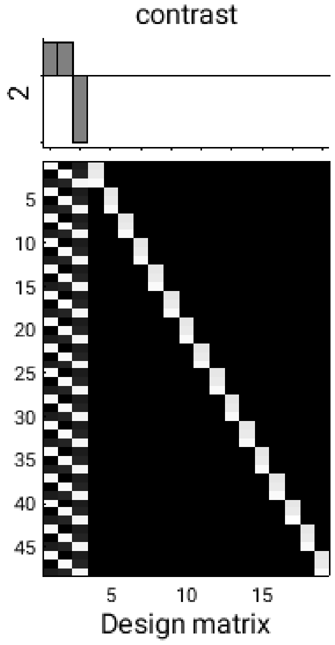

SPMresults: /meg/TFStats/PhsStats/eeg
Height threshold T = 3.385185 {p<0.001 (unc.)}
Extent threshold k = 0 voxels

Statistics: ***p-values adjusted for search volume***

cluster-level				peak-level					Hz ms
$p_{FWE-corr}$	$q_{FDR-corr}$	k_E	p_{uncorr}	$p_{FWE-corr}$	$q_{FDR-corr}$	T	$(Z_{\equiv})$	p_{uncorr}	
0.000	0.000	53	0.000	0.030	0.042	4.77	4.08	0.000	16 180 1

table shows 3 local maxima more than 8.0mm apart

Height threshold: T = 3.39, p = 0.001 (0.518)
Extent threshold: k = 0 voxels
Expected voxels per cluster, <k> = 6.098
Expected number of clusters, <c> = 0.73
FWEp: 4.554, FDRp: 4.769, FWEc: 53, FDRc: 53

Degrees of freedom = [1.0, 30.0]
FWHM = 4.7 35.3 Hz ms ; 4.7 7.1 {voxels}
Volume: 21175 = 4235 voxels = 123.4 resels
Voxel size: 1.0 5.0 Hz ms ; (resel = 33.07 voxels)

Appendix 2. fMRI Preprocessing and Statistics

We will keep description of the preprocessing and analysis of the fMRI data to a minimum, given that fMRI analysis is not the subject of the present research topic and paper. We start with preprocessing Subject 15's data. For the fMRI experiment, there were 9 runs (sessions), rather than the 6 in the M/EEG experiment. This is because the stimulus onset asynchrony was increased in the fMRI experiment, by virtue of jittering, which is necessary to estimate the BOLD response versus interstimulus baseline (e.g., to compare with the evoked EEG/MEG response versus prestimulus baseline). Preprocessing involves the following modules, which can be found under the menu "**SPM – Spatial**":

A2.1 Coregistration of EPI (fMRI) data

The first step is to coregister the 9 runs of 208 images to one another (i.e., correcting for movement) using a rigid-body transform. Select "**Realign: Estimate & Reslice**", and on the "Data" item, add nine new "Sessions". Then, for the first session, select the "`*_run-01_bold.nii`" image in the "`func`" directory of Subject 15, and then repeat for the remaining eight sessions. On the "Reslice Options" item, change the default value of "All Images + Mean Image" to "Mean Image Only". This is because we do not re-slice the EPI data: the coregistration parameters will be stored in the header information of the NIFTI file, and can be combined with the normalisation parameters when re-slicing the normalised images below (re-slicing can introduce interpolation artifacts so generally best to reduce number of re-slicings). However, we do need a resliced mean image, which we can use for coregistration with the T1 below. Thus the contents of the "`*_bold.nii`" files will change (as header updated), but no new files will be created, except for the "`mean*.nii`" file.

A2.2 Segmentation of T1 images

We will use SPM12's unified segmentation to estimate normalisation parameters to MNI space (this is generally better that SPM's old normalisation, though an alternative would be to normalise the mean EPI image to an EPI template, which, even though lower spatial resolution, at least contains the same distortions). Select "**Segment**" item, and for the "Volumes" item, select the "`sub-15_ses-mri_acq-mprage_T1w.nii`" image in the "`anat`" directory of Subject 15. You can leave the remaining menu items at their defaults (even though the tissue segment images are not required in this analysis), but you do need to change the final option "Deformation Fields" from "None" to "Forward" (these are the normalisation parameters we will apply to EPI data below).

A2.3 Coregistration of mean EPI (fMRI) to T1 (sMRI)

Because we have determined the normalisation parameters from the subject's native space to MNI space via the unified segmentation of their T1 image, we need to coregister all the EPI images to that T1 image, so that the normalisation warps can be later applied. Select "**Coregister: Estimate**" item, and for the "Reference Image", select the same "`sub-15_ses-mri_acq-mprage_T1w.nii`" image in the "`anat`" directory. For the "Source Image", select "Dependency" and then use Ctrl and Shift to select all 9 sessions from the "Realign" stage.

A2.4 Application of Normalisation parameters to EPI data

We can now apply the normalisation parameters (warps) to each of the EPI volumes, to produce new, re-sliced "`w*`" images. Select "**Normalise – Write**" item, and for the "Deformation Field", use select the "Segment: Forward Deformations" dependency. For the "Images to Write", select the "Coregister: Estimate: Coregistered Images". You can also change the default voxel size from [2 2 2] to [3 3 3] since the original data are [3 3 3.9] (for Subject 15).

A2.5 Smoothing

Finally, we smooth the normalised images by an 8mm isotropic Gaussian kernel to produce "`sw*`" images. So select the "**Smooth**" item, and select the input to depend on the output of the prior Normalisation stage.

Save batch and review

You can save this batch file (it should look like the "`batch_preproc_fmri_job.m`" file in the "`code/scripted`" directory), and then run it. You can inspect the realignment parameters, normalisations, etc. as described in other chapters of the SPM manual. Make a new directory called "`Stats`" in the "`Sub15/func`" directory.

A2.6 Creating a 1st-level (fMRI) GLM

You first need to create a MATLAB file that contains the onsets, durations and names of every trial in each run for each subject. This information is stored in a "`*.tsv`" file of the BIDS format, and will be used for the "Multiple Conditions" entry below.

```
% Create SPM's multiple conditions files
%-----------------------------------------------------------------------
runs = spm_BIDS(BIDS,'runs', 'modality','func', 'type','bold', 'task','facerecognition');
nrun = numel(runs);

trialtypes = {'Famous','Unfamiliar','Scrambled'}; % impose order
for s = 1:nsub
    for r = 1:nrun
        d = spm_load(char(spm_BIDS(BIDS,'data',...
            'modality','func','type','events','sub',subs{s},'run',runs{r})));
        clear conds
        for t = 1:numel(trialtypes)
            conds.names{t} = trialtypes{t};
            conds.durations{t} = 0;
            conds.onsets{t} = d.onset(strcmpi(d.stim_type,trialtypes{t}));
        end
        save(fullfile(outpth,subdir{s},'func',...
            sprintf('sub-%s_run-%s_spmdef.mat',subs{s},runs{r})),'-struct','conds');
    end
end
```

Select the "**fMRI model specification**" option from the "SPM – Stats" menu. Select the new "`Stats`" directory you created as the output directory. Set the "Units for design" to seconds (since our onsets files are in units of seconds) and the "interscan interval" (TR) to "2". Then under the "Data & Design" option, create a new Session, and then select all the "`sw*_run-01_bold.nii`" images in the "`func`" directory as the "Scans". Then under the "Multiple conditions" option, press "Specify" and select the file "`sub-15_run-01_spmdef.mat`" that was created above. Then under the "Multiple regressors" option, press "Specify" and select the file matching "`rp*.txt`" in the "`func`" directory. This is a text file that contains the 6

movement parameters for each scan, which was created during Realignment above, and we will add to the GLM to capture residual motion-related artifacts in the data.

For the basis functions, keep "Canonical HRF", but change the "model derivatives" from "no" to "time and dispersion derivatives" (see earlier Chapter manuals). Then keep the remaining options as their defaults.

You then need to replicate this for the remaining 8 sessions, updating all three fields each time: i.e., the scans, conditions and (movement) regressors. (It is at this point, that you might want to switch to scripting, which is much less effort!). Leave the remaining options as their defaults.

A2.7 Model Estimation

Add a module for "**Model Estimation**" from the Stats bar and define the file name as being dependent on the results of the factorial design specification output.

A2.8 Setting up contrasts

To create some contrasts, select "**Contrast Manager**" from the Stats bar. Define the file name as dependent on the model estimation. The first contrast will be a generic one that tests whether significant variance is captured by the 3 canonical HRF regressors (one per condition). So create a new F-contrast, call it the "Canonical HRF effects of interest", and enter as the weights matrix:

[1 0 0 0 0 0 0 0 0 0 0 0 0 0 0
 0 0 0 1 0 0 0 0 0 0 0 0 0 0 0
 0 0 0 0 0 0 1 0 0 0 0 0 0 0 0]

Then select "Replicate" to reproduce this across the 9 sessions. We can also define a T-contrast to identify face-related activation, e.g., "Faces > Scrambled Faces", given the weight matrix [0.5 0 0 0.5 0 0 -1 0 0 0 0 0 0 0 0], again replicated across sessions. (Note that there are 3 basis functions per condition, and the zeros here ignore the temporal and dispersion derivatives, but if you want to include them, you can add them as separate rows and test for any face-related differences in BOLD response with an F-contrast).

Finally, for the group-level (2nd-level) analyses below, we need an estimate of activation of each condition separately (versus baseline), averaged across the sessions. So create three new T-contrasts, whose weights correspond to the three rows of the above F-contrast, i.e., that pick out the parameter estimate for the canonical HRF for Famous, [1 0 0 0 0 0 0 0 0 0 0 0 0 0 0]; for Unfamiliar [0 0 0 1 0 0 0 0 0 0 0 0 0 0 0]; and for Scrambled [0 0 0 0 0 0 1 0 0 0 0 0 0 0 0]. These T-contrasts will be numbered 3-5, and used in group analysis below.

Save batch and review

You can save this batch file (it should look like the "`batch_stats_fmri_job.m`" file in the "`code/scripted`" directory). When it has run, you can press "**Results**" from the SPM Menu window, select the "`SPM.mat`" file in the "BOLD" directory, and explore some of the contrasts. However, we will wait for the group analysis below before showing results here.

Creating a script for analysis across subjects

Now that we have created a pipeline for fMRI preprocessing and analysis for a single subject, we can script it to run on the remaining 15 subjects. Below is an example from the "`master_script.m`":

```
spm_jobman('initcfg');
spm('defaults', 'fmri');

parfor (s = 1:nsub, numworkers)
    % Change to subject's directory
    cd(fullfile(outpth,subdir{s},'func'));

    % Preprocessing
    %-----------------------------------------------------------------------
    jobfile = {fullfile(scrpth,'batch_preproc_fmri_job.m')};
    inputs  = cell(nrun+2,1);
    for r = 1:nrun
        inputs{r} = cellstr(spm_select('FPList',fullfile(outpth,subdir{s},'func'),...
            sprintf('^sub-.*run-%s_bold\\.nii',runs{r})));
    end
    inputs{nrun+1} = cellstr(spm_select('FPList',fullfile(outpth,subdir{s},'anat'),...
        '^sub-.*_T1w\.nii$'));
    inputs{nrun+2} = cellstr(spm_select('FPList',fullfile(outpth,subdir{s},'anat'),...
        '^sub-.*_T1w\.nii$'));
    spm_jobman('run', jobfile, inputs{:});

    % First-level statistics
    %-----------------------------------------------------------------------
    jobfile = {fullfile(scrpth,'batch_stats_fmri_job.m')};
    inputs  = {}; %cell(nrun*3+1,1);
    inputs{1} = {fullfile(outpth,subdir{s},'func','Stats')};
    for r = 1:nrun
        inputs{end+1} = cellstr(spm_select('FPList',fullfile(outpth,subdir{s},'func'),...
            sprintf('^swsub-.*run-%s_bold\\.nii$',runs{r})));
        inputs{end+1} = cellstr(fullfile(outpth,subdir{s},'func',...
            sprintf('sub-%s_run-%s_spmdef.mat',subs{s},runs{r})));
        inputs{end+1} = cellstr(spm_select('FPList',fullfile(outpth,subdir{s},'func'),...
            sprintf('^rp.*run-%s.*\\.txt$',runs{r})));
    end
    spm_jobman('run', jobfile, inputs{:});

    if ~keepdata
        f = cellstr(spm_select('FPList',fullfile(outpth,subdir{s},'func'),'^sub-.*_bold.*'));
        spm_unlink(f{:});
        f = cellstr(spm_select('FPList',fullfile(outpth,subdir{s},'func'),'^w.*_bold.*'));
        spm_unlink(f{:});
    end
end
```

Once you have run this script, we can do 2nd-level (group) statistics on resulting contrast images for each condition (averaged across 9 runs).

A2.9 Group Statistics on fMRI data

Now we have a new set of 16x3 NIfTI images for each subject and each condition, we can put them into the same repeated-measures ANOVA that we used to test for differences in power across sensors in the time-frequency analysis above, i.e., re-use the "`batch_stats_rmANOVA_job.m`" file created above. This can be scripted as:

```
% fMRI stats across subjects
%-----------------------------------------------------------------------
jobfile = {fullfile(scrpth,'batch_stats_rmANOVA_job.m')};
inputs  = cell(nsub+1, 1);
inputs{1} = {fullfile(outpth,'func')};
for s = 1:nsub
```

```
    % Assumes that these T-contrasts in fMRI 1st-level models are famous, unfamiliar,
    % scrambled (averaged across sessions)
    inputs{s+1,1} = cellstr(spm_select('FPList',fullfile(outpth,subdir{s},'func','Stats'),...
        '^con_000[345]\.nii$'));
end
spm_jobman('run', jobfile, inputs{:});
```

Save batch and review

When the script has run, press "**Results**" from the SPM Menu window and select the "`SPM.mat`" file in the "`func`" directory. From the Contrast Manager window, select the pre-specified T-contrast "Faces (Fam+Unf) > Scrambled". Within the "Stats: Results" window, when given the option, select the following: Apply Masking: None, P value adjustment to control: FWE, keep the threshold at 0.05, extent threshold {voxels}: 30; Data Type: Volumetric 2D/3D. The Graphics window should then show what is in Figure A2.1 below. Note the left and right occipital face area (OFA) clusters and right fusiform face area (FFA) cluster.

In the main analysis, we can use these three clusters as priors for constraining the inversion of our EEG/MEG data. To do this, we need to save these as an image. Press the "save…" button in the bottom right of the SPM Results window, and select the "all clusters (binary)" option. The window will now prompt you for an output filename, in which you can type in "`spmT_0002_05cor.nii`". This image will be output in the "`func`" SPM stats directory, and will be used in the Group and fMRI Optimized Source Reconstruction section of the main paper.

Faces (Fam+Unf) > Scrambled

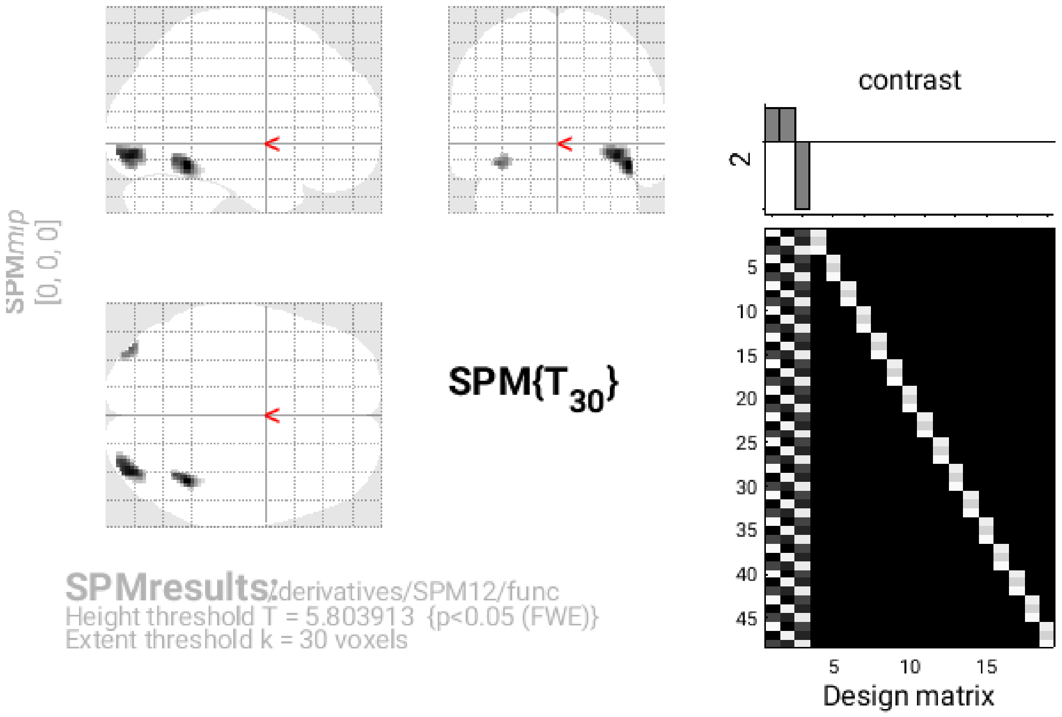

Statistics: ***p-values adjusted for search volume***

set-level		cluster-level				peak-level					mm mm mm
p	c	$p_{FWE\text{-}corr}$	$q_{FDR\text{-}corr}$	k_E	p_{uncorr}	$p_{FWE\text{-}corr}$	$q_{FDR\text{-}corr}$	T	$(Z_{\equiv})$	p_{uncorr}	
0.000	3	0.000	0.000	120	0.000	0.000	0.005	8.91	6.18	0.000	42 -52 -14
		0.000	0.000	180	0.000	0.000	0.005	8.65	6.08	0.000	36 -86 -10
		0.000	0.014	37	0.008	0.001	0.034	7.52	5.60	0.000	-38 -86 -14

table shows 3 local maxima more than 8.0mm apart

Height threshold: T = 5.80, p = 0.000 (0.050)
Extent threshold: k = 30 voxels, p = 0.016 (0.001)
Expected voxels per cluster, <k> = 4.709
Expected number of clusters, <c> = 0.00
FWEp: 5.804, FDRp: 7.523, FWEc: 5, FDRc: 37

Degrees of freedom = [1.0, 30.0]
FWHM = 13.0 12.9 12.6 mm mm mm; 6.5 6.4 6.3 {voxels}
Volume: 1515832 = 189479 voxels = 671.8 resels
Voxel size: 2.0 2.0 2.0 mm mm mm; (resel = 261.76 voxels)

Figure A2.1. Group SPM for Faces vs Scrambled fMRI data thresholded for clusters with at least 30 voxels that survive p<.05 FWE-corrected, resulting in a right fusiform face area (FFA) and left and right occipital face area (OFA).
